# Exploring genomic analysis and methylome profiling in longitudinal series of p.G12C KRAS mutated NSCLC patients treated with sotorasib

**DOI:** 10.1016/j.jlb.2026.100467

**Published:** 2026-04-27

**Authors:** Francesco Pepe, Francesco Passiglia, Claudia Scimone, Gianluca Russo, Giuseppina Roscigno, Domenico Cozzolino, Angela Listì, Caterina De Luca, Edoardo Garbo, Luisella Righi, Viola Calabrò, Caroline Lacoux, Paul Hofman, Silvia Novello, Giancarlo Troncone, Umberto Malapelle

**Affiliations:** a Department of Public Health, Federico II University of Naples, Via S. Pansini, 5, 80131, Naples, Italy; b Department of Oncology, University of Turin, S. Luigi Gonzaga Hospital, Orbassano, TO, Italy; c Department of Biology, Complesso Universitario Monte Sant'Angelo, University of Naples Federico II, Via Cintia 4, 80126, Naples, Italy; d DepIHU RespirERA Laboratory of Clinical and Experimental Pathology, FHU OncoAge, Biobank BB 0033-00025, University Côte d’Azur Nice France, France

**Keywords:** Genomic analysis, Methylation profile, Liquid biopsy, Lung cancer, Target treatment

## Abstract

in the genomic era, the advent of next generation sequencing (NGS) technologies has rapidly transformed the clinical paradigm of NSCLC patients who could benefit from a wide series of clinically approved biomarker driven therapies. Among them, *KRAS* p. G12C hotspot mutation became part of the mandatory testing gene panel by electing NSCLC patients to sotorasib. Epigenomic signatures, including hypermethylation of CpGs islands, may be relevant in tailoring therapeutic algorithms in oncogene addicted NSCLC patients. Here we aimed to dynamically track *KRAS* p. G12C genomic variations by integrating methylation profile in a longitudinal series of n = 91 liquid biopsy samples from n = 22 p. G12C positive NSCLC patients treated with sotorasib. A combined NGS panel (Avida Duo Methyl Reagent Kit, Avida Biomed) simultaneously evaluating n = 105 cancer-related genes and calculating methylation index (MI) score among 3400 differentially methylated regions (DMRs) was adopted, correlating molecular data with clinical outcomes. Overall, exon 2 p. G12C *KRAS* mutation was detected in 40.9%, 15.8% and 70.6% baseline, T1 and TP samples, respectively. MI was successfully measured in all instances. Of note, exon 2 p. G12C *KRAS* mutation and MI score highlighted a trend simultaneously moving forward T1 point (r = 0.68, *p* = 0.06) and TP (r = 0.87, p = 0.000103). Methylation signature may be combined with genomic analysis to personalize therapeutic strategies for *KRAS* p. G12C mutated NSCLC patients. “Multiomic” analysis of tumor-informative molecular targets (genomic profile, methylation status) lay the basis for dynamic fingerprints of NSCLC patients preventing early relapses and augmenting clinical benefits of targeted therapies.

## Graphical abstract

**Figure:**
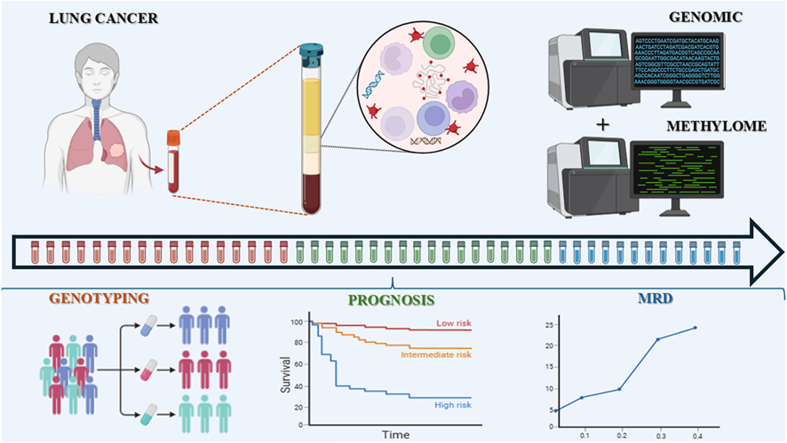

## 1 Introduction

Lung cancer (LC) represents one of the most leading causes of death worldwide across solid malignancies [1]. Conventional diagnostic procedures are affected by the lack of analytical sensitivity and specificity to early detect LC patients drastically impacting on the transition to advanced stage (IIIB-IV) [2]. In the last decade, precision medicine has revolutionized the clinical paradigm of advanced non-small cell lung cancer (NSCLC) patients [3]. Particularly, guidelines from CAP/AMP/IASCL societies established a panel of mandatory testing genes able to identify oncogene-addicted patients sensitive to targeted therapies [4]. This panel includes DNA-based [(*EGFR* (Epidermal Grow Factor Receptor), *BRAF* (v-Raf murine sarcoma viral oncogene homolog B), HER-2 (Human Epidermal growth factor Receptor 2)] and RNA-based [(*ALK* (anaplastic lymphoma kinase), *ROS1* (protooncogene 1 receptor tyrosine kinase), *RET* (Proto-Oncogene Tyrosine-Protein Kinase Receptor Ret), *NTRK* (Neurotrophic Tyrosine Receptor Kinase) and *MET* (MET Proto-Oncogene, Receptor Tyrosine Kinase)] biomarkers crucial for the therapeutic management of NSCLC patients [5,6]. Recently, exon 2 p. G12C *KRAS* mutation emerged as a novel therapeutic target identifying NSCLC patients eligible to selective covalent inhibitors, like sotorasib and adagrasib [7]. In addition, *NRG1* (Neuregulin 1) has been accelerating to stratify NSCLC patients to novel mABs [8]. Not surprisingly, both innate and acquired resistance mechanisms significantly reduce the clinical benefit of *KRAS* p. G12C covalent inhibitors in NSCLC cases [9]. Novel molecular hallmarks should be identified to optimize the clinical stratification of *KRAS* p. G12C mutant advanced NSCLC patients [10,11]. It has been demonstrated that specific patterns of epigenetic alterations including DNA methylation, histone modification, and the aberrant expression of non-coding RNA, are recurrent in NSCLC playing a pivotal role in tumor progression potentially impacting on clinical outcomes [[12], [13], [14]]. Due tothe low abundance of circulating tumor DNA (ctDNA) in torrent blood of NSCLC patients, multi-omics analysis integrating tumor addicted genomic alterations and methylation signatures may act as potential tool for guiding clinical decision-making procedures in advanced NSCLC patients [15,16]. Here, we sought to evaluate technical and clinical performance of a combined genomic plus methylome NGS analysis on a longitudinal series of liquid biopsy specimens from *KRAS* p. G12C mutant advanced NSCLC patients receiving sotorasib in the real-world scenario.

## 2 Methods

### 2.1 Participants

**Supplementary Fig. 1:**
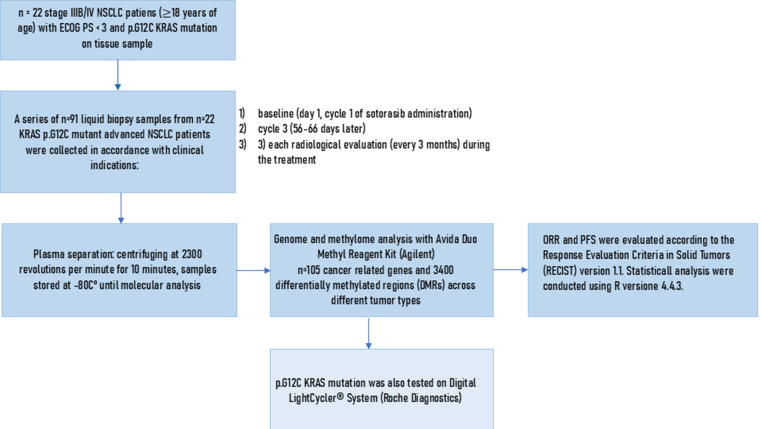
Flowchart diagram of NSCLC patients enrolled in the trial.

Patients (≥18 years of age) with ECOG PS < 3 and p. G12C *KRAS* mutation on tissue sample, receiving a diagnosis of stage IIIB-IIIC/IV NSCLC (according to the eighth version of the American Joint Committee on Cancer/International Association for the Study of Lung Cancer tumor‐node‐metastasis [TNM] staging system) on histological or cytological samples were enrolled. All patients relapsed from at least one line of previous therapy before receiving sotorasib (960 mg orally once daily) until progression or unacceptable toxicity; participated to the PROMOLE translational study at the Department of Oncology of the University of Turin (Italy) and signed and dated the Informed Consent & privacy Form (ICF). Clinical, pathologic, and molecular data as well as treatment efficacy/tolerability outcomes were retrieved from the electronic medical repository archived at the University of Turin. The radiologic examination was performed as follows: computed tomography scans were approached at baseline, at week 12, and every 12 weeks of therapy until disease progression. Clinical responses were defined in accordance with RECIST version 1.1. Written informed consent was acquired from all patients and documented according to “The Italian Data Protection Authority” (http://www.garanteprivacy.it/web/guest/home/docweb/-/docwebdisplay/export/2485392). All information regarding human material was managed using anonymous numerical codes and all samples were handled in compliance with the Helsinki Declaration (http://www.wma.net/en/30publications/10policies/b3/). The PROMOLE protocol was previously approved by the Independent Ethic Committee of S. Luigi Hospital, University of Turin (ethics approval number 73/2018 of 2024.01.30) (Supplementary Fig. 1).

### 2.2 Liquid biopsy collection and management

Peripheral blood samples (ranging from two to eight collecting points) were withdrawn in accordance with clinical indication: 1) baseline (day 1, cycle 1 of sotorasib administration); 2) cycle 3 (56-66 days later); 3) each radiological evaluation (every 3 months) during the treatment. A longitudinal series of n = 91 liquid biopsy samples collected from n = 22 *KRAS* p. G12C mutant advanced NSCLC patients were retrieved from internal archive of University of Turin-San Luigi Hospital and shipped to the Cytopathology and Predictive Molecular Pathology Unit at University of Naples Federico II. Overall, two aliquots (containing two ml of plasma) were available for each clinically relevant time point. Plasma was separated centrifuging blood at 2300 revolutions per minute for 10 min, in accordance with standardized handling procedures and stored at −80C° until the shipment. Circulating-free DNA (cfDNA) was automatically purified from 2 ml of plasma samples adopting QIAsymphony instrument (Qiagen) equipped with the QIAsymphony DSPVirus/Pathogen Midi Kit, following previously validated protocol [17]. (**Supplementary file 1**) Finally, cfDNA was resuspended in 60 μl of DNAse and RNAse-free water (Thermo Fisher Scientifics, Waltham, MA, USA) and stored in dedicated tubes at −80C° until molecular analysis.

### 2.3 cfDNA evaluation in liquid biopsy samples

Before molecular analysis, cfDNA abundance in liquid biopsy samples was calculated. Briefly, 2 μl of extracted nucleic acids were automatically dispensed into Cell-free DNA ScreenTape (Agilent) equipped on TapeStation 4200 (Agilent) microfluidic platform, following manufacturer procedures. Proprietary software measured cfDNA abundance in biological samples comparing between 170 bp and high molecular weight (HMW) DNA >700 bp peaks.

### 2.4 Molecular analysis and methylation index (MI) calculation

A combined genomic and methylome NGS panel (Avida DNA Expanded Cancer and Avida Methyl 3400 DMR Cancer panels prepared with Avida Duo Methyl Reagent Kit, Agilent) was implemented to evaluate genomic alterations and measure methylation index (MI) in longitudinal series of liquid biopsy samples. It has been demonstrated that methylation profile significantly correlated with tumor burden. Briefly, MI analysis was set to longitudinally detect tumor related signatures tracking tumor burden across longitudinal points [18]. This panel simultaneously analyzes molecular alterations in n = 105 cancer related genes and 3400 differentially methylated regions (DMRs) across different tumor types distinguishing between tumor patients and healthy subjects [18]. The Avida hybrid capture technology is built on a proprietary design of three-dimensional, biotinylated anchor probes scaffolding DNA regions and stabilizing interaction with target sites. Trimmed Unique molecular identifier (UMI) counts removing single strand duplicates and low-quality reads may increase technical sensitivity and specificity of Avida Duo Methyl Reagent Kit up to 94.0% and >98.0%, respectively, starting from 5.0 to 10.0 ng of input.

Of note, 1-100 ng of input at 170 ± bp was required to perform molecular analysis and calculate MI. Briefly, two consecutive target captures yielded targeted sequencing (TS) and targeted methylation sequencing (TMS). A sequential approach generating TMS libraries (based on bisulfite conversion) from unhybridized TS libraries was used to generate template (including n = 24 matched TS and TMS libraries) in accordance with manufacturer instructions. Libraries were diluted at 2-4 nM and pooled together unbalancing TS and TMS libraries 2.5:1 in accordance with manufacturer procedures. Finally, TS and TMS pooled libraries were sequenced on NextSeq 550 Dx platform (Illumina, San Diego, USA) following manufacturer instructions. FASTQ files were manually uploaded on Alissa Report software (v 2.0.0) (Agilent Technologies) where genomic and methylation data were automatically carried out adopting proprietary bioinformatic pipelines. Briefly, genomic and methylome data were filtered by inspecting mandatory (total number of raw reads >200.000, total number of raw bases >15.000.000, average read length forward and reverse >40) and recommended (mean insert size >120, fraction of inaccessible targeted bases <0.02, fraction of targeted bases at 100x coverage >0.6) technical parameters. As regards TMS analysis pipeline, CpGs calculated from CpG (5′C-phosphate-G-3′) and CHX methylation data from the cytosine (C) to thymine (T) and guanine (G) to adenine (A) conversion were automatically inspected by analysis software. MI was measured using a proprietary bioinformatic algorithm able to identify tumor methylated CpGs (mCpGs) counting CHH on target methylation profile (<17.5). Methylation profile was also analyzed adopting early access SomaMethyl bioinformatic pipeline from SeqOne Genomics (Montpellier, France) able to automatically calculate MI by proprietary algorithm. A training set of n = 14 previously tested NSCLC patients (n = 7 positive and n = 7 negative for MI scoring) was uploaded to set up threshold (≥1) for MI scoring.

Moreover, p. G12C *KRAS* mutation was also longitudinally evaluated on Digital LightCycler® System (Roche Diagnostics) in accordance with manufacturer procedures. A total of 5.0 μl of cfDNA was manually combined with parameter specific reagents (PSR) (containing premixed, dried p. G12C *KRAS* primers and probes) and Digital LightCycler® master mix (Roche Diagnostics), then loaded into the Digital LightCycler® universal plate achieving 28.000 partitions in each well. (Roche Diagnostics). Moreover, cfDNA fragments were automatically partitioned adopting Digital LightCycler® Partitioning Engine platform (Roche Diagnostics) following technical instructions [19]. Up to n = 12 plates can be simultaneously processed by Digital LightCycler® System (Roche Diagnostics). Moreover, technical parameters including total valid partitions, positive partitions and number of copies/μl for mutant (FAM) and wild type (HEX) signal, were automatically calculated by proprietary software (Digital LightCycler® System Development Software) correlating p. G12C positive signal with copies/μl of mutant fragments. In addition, molecular p. G12C status “positive or negative” was automatically called by proprietary software.

### 2.5 Statistical analysis

All statistical analyses on technical parameters and molecular records were conducted using Prism GraphPad software, version 10.0 for Windows (GraphPad Software, San Diego, CA, USA; www.graphpad.com) performing either Student's t (for two variables) or one-way ANOVA (for multiple variables) test followed by Tukey's post hoc test setting ∗ p < 0.05 as a cut-off for statistical significance. Pearson correlation coefficients were calculated to assess the linear relationship between variables by using GraphPad Prism. Briefly, evaluating r value, the corresponding p-values were measured, with ∗∗∗p < 0.001 cut-off for statistical significance.

Moreover, clinical, pathologic, and molecular characteristics of participants treated with sotorasib were summarized either by descriptive statistics or as categorical tables. Analysis was performed, including means, standard deviations, medians, quartiles, and absolute/relative frequencies (with their respective two‐sided 95% confidence interval [CI] limits, where relevant), according to the specific variables. Comparisons of continuous variables among groups were approached using the Wilcoxon rank-sum or Kruskal-Wallis tests. Categorical variables were compared using Fisher's exact or chi-squared tests. Overall response rate (ORR) and progression-free survival (PFS) were evaluated according to the Response Evaluation Criteria in Solid Tumors (RECIST) version 1.1. PFS was defined ranging from therapy initiation to documented disease progression or death. Patients without progression were censored at the date of the last imaging assessment demonstrating no progression. Overall survival (OS) ranged between the immune checkpoint inhibitor (ICI) initiation to death from any cause. Survival outcomes were estimated using the Kaplan–Meier method and compared with the log-rank test, by using a p value < 0.05 as threshold for statistical significance. All analyses were conducted using R version 4.4.3.

## 3 Results

### 3.1 Patients’ characteristics

Between November 2020 and December 2022, a total of n = 22 *KRAS* p. G12C mutant advanced NSCLC patients receiving sotorasib were enrolled. Baseline clinical characteristics are summarized in Table 1. The median age was 70.5 years (range, 46–81), and 52.2% of patients were male. Most patients had an ECOG performance status (PS) of 0 (63.6%), while a history of tobacco exposure and histological diagnosis of lung adenocarcinoma were assessed in all instances. PD-L1 expression was ≥50% in 22.7% of cases, 1–49% in 45.5%, and <1% in 31.8%. Brain metastases were the most frequent metastatic site (36.4%). Moreover, 63.6% of patients had previously received anti–PD-L1 therapy. All patients were evaluated for tumor response assessment: 7 (31.8%) experienced a partial response (PR), 11 (50%) had stable disease (SD), and 4 (18.2%) had progressive disease (TP) as their best response to sotorasib. The median follow-up calculated with the reverse Kaplan-Meier was 32 months (range 2-35) for the overall cohort at the time of data cut-off (Table 1).

**Table 1:** Methylation Index according to patients’ characteristics.

Patients' Characteristics	Methylation Index	P-value
	Median [Min, Max]	
**Age**		
<75y (N = 16)	0.636 [0.0800, 14.8]	0.641
≥75y (N = 6)	0.564 [0.130, 3.30]	
**ECOG-PS**		
0 (N = 14)	0.653 [0.0800, 14.8]	0.441
1-2 (N = 8)	0.474 [0.130, 12.3]	
**Smoking history**		
<30p/y (N = 8)	0.666 [0.08, 14.8]	0.492
≥30p/y (N = 11)	0.589 [0.130, 2.13]	
Missing (N = 3)		
**Brain metastases**		
Yes (N = 8)	0.548 [0.0800, 12.3]	0.815
No (N = 14)	0.653 [0.130, 14.8]	
**PD-L1 TPS**		
PD-L1 Negative (N = 17)	0.555 [0.08, 12.3]	0.164
PD-L1 Positive (N = 5)	3.30 [0.366, 14.8]	
**Previous Immunotherapy**		
Yes (N = 14)	0.747 [0.0800, 14.8]	0.441
No (N = 8)	0.539 [0.13, 12.3]	
**Best Response**		
Responders (N = 14)	0.932 [0.409, 14.5]	0.123
Non-Responders (N = 8)	0.540 [0.0800, 14.8]	

*Abbreviations*: ECOG-PS (*Eastern Cooperative Oncology Group - Performance Status*); PD-L1 (*Programmed Death-Ligand 1*); TPS (*Tumor Proportion Score*).

### 3.2 cfDNA abundance in liquid biopsy samples

Overall, cfDNA measurement was successfully carried out in all instances. Of note, cfDNA score was clearly inspected in 78 out of 91 cases (85.7%) achieving a median value of 73.0% ranging from 32.0% to 93.0%. Baseline and TP samples highlighted a median cfDNA score of 72.0% (from 53.0% to 83.0%) and 77.0% (from 57.0% to 91.0%). In addition, a median of 0.2 ng/μl (from 0.1 to 2.4 ng/μl) was inspected. In detail, baseline and TP samples revealed a median of 0.2 ng/μl (from 0.1 ng/μl to 0.8 ng/μl) and 0.3 ng/μl (from 0.1 ng/μl to 1.3 ng/μl), respectively (Supplementary Table 1).

### 3.3 Genomic and methylome data

**Supplementary Fig. 2:**
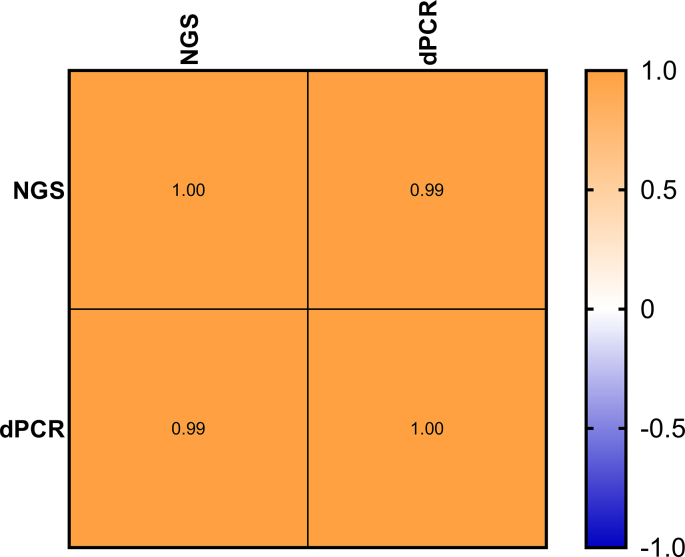
The heatmap highlights the Pearson correlation coefficients between NGS and dPCR detecting p.G12C *KRAS* mutation. The r value indicates a high correlation between the two analytical methods (r = 0.99). The color gradient reflects the magnitude and direction of the correlation, ranging from blue (negative) to orange (positive). The correlation is statistically significant (p-value of 6.91 × 10^−77^).

Genomic analysis and methylation index were evaluated in all instances. In particular, an average of 12460161.9 (ranging from 5139660.0 to 45165008.0) total number of raw reads, of 3052515.0 (ranging from 458720.0 to 11360524.0) number of mapped reads; of 2949844.9 (ranging from 424865.0 to 11077138.0) high quality reads; of 434.7 (ranging from 63.0 to 1617.0) median depth in targeted region were identified among genomic data. Moreover, an average of 15596177.5 (ranging from 2247880.0 to 47794630.0) total number of raw reads, of 6932619.7 (ranging from 107636.0 to 15259654.0) number of mapped reads, of 6330388.8 (ranging from 81958.0 to 13361940.0) high quality reads, a median of 10486874,43 (ranging from 135213.0 to 95510093.0) total number of analyzed cytosines, of 11.8 (ranging from 5.3 to 27.5) methylated C in CpG context, of 4.2 (ranging from 0.5 to 18.0) methylated C in CHG context, of 3.7 (ranging from 0.5 to 13.5) methylated C in CHH was identified among methylation data (Supplementary Table 2 A-B). Considering a technical cut-off of 0.2%, exon 2 p. G12C *KRAS* mutation was successfully identified in 9 out of 22 (40.9%) and 12 out of 17 (70.6%) baseline and TP samples, respectively, showing a median variant allele fraction (VAF) of 9.4% (from 0.7% to 44.2%) and 19.2% (from 0.2% to 65.4%), respectively (Table 2). In addition, dPCR system successfully analyzed all samples highlighting a median VAF of 18.6% (from 0.2% to 76.7%) in 27 out of 91 (29.7%) in p. G12C positive samples (Supplementary Table 3). A median VAF of 11.6% (ranging from 0.4% to 56.5%) and 25.9% (ranging from 0.2% to 76.7%) was measured in basal and TP samples, respectively. A statistically significant correlation was yielded comparing NGS and dPCR analysis (≥2 positive partitions). (r = 099, p-value of 6.91 × 10^−77^) (Supplementary Fig. 2).

**Table 2:** Schematizing report of Methylation index and *KRAS* p.G12C VAF across longitudinal plasma samples.

ID Sample	Collection point	Methylation Index	*KRAS* p.G12C (VAF%)	ID Sample	Collection point	Methylation Index	*KRAS* p.G12C (VAF%)
**ID01**	T_0_	1.7	ND	**ID12**	T_0_	0.5	ND
	T_1_	0.4	ND		T_1_	0.3	ND
	Tp	0.3	0.6		T_2_	0.1	ND
**ID02**	T_0_	12.3	44.2		T_3_	0.1	ND
	T_1_	4.6	43.1		T_4_	0.5	ND
	Tp	7.4	65.4		T_5_	0.1	ND
**ID03**	T_0_	14.5	ND		T_6_	0.3	ND
	T_1_	1.5	ND	**ID13**	T_0_	0.1	ND
	T_2_	2.4	ND		T_1_	0.3	ND
	Tp	5.6	ND		T_2_	0.9	ND
**ID04**	T_0_	0.2	ND		T_3_	0.5	0.2
	T_1_	1.3	ND		T_4_	0.5	ND
**ID05**	T_0_	0.5	ND		T_5_	1.2	ND
	T_1_	0.2	ND		Tp	2.0	1.0
	T_2_	0.3	ND	**ID14**	T_0_	0.1	ND
	T_3_	0.6	ND		T_1_	0.3	ND
	T_4_	0.7	ND		Tp	0.3	ND
	T_5_	0.7	ND	**ID15**	T_0_	0.6	1.1
	T_6_	0.6	0.6		T_1_	0.2	ND
	T_7_	0.4	1.1		T_2_	0.7	ND
**ID06**	T_0_	0.4	ND		Tp	0.3	ND
	T_1_	0.1	ND	**ID16**	T_0_	0.9	ND
	T_2_	0.4	ND		T_1_	0.4	ND
	Tp	0.1	ND		T_2_	3.0	0.9
**ID07**	T_0_	0.3	ND		T_3_	12.9	22.7
	T_1_	0.2	ND		Tp	55.8	64.8
	T_2_	0.2	1.1	**ID17**	T_0_	0.4	ND
	Tp	0.1	4.3		Tp	0.3	ND
**ID08**	T_0_	0.1	ND	**ID18**	T_0_	0.6	3.2
	T_1_	0.1	ND		T_1_	0.2	ND
	T_2_	0.1	ND		Tp	0.5	14.8
	T_3_	0.1	ND	**ID19**	T_0_	0.7	6.4
	T_4_	0.3	ND		Tp	1.8	23.1
	T_5_	0.4	ND	**ID20**	T_0_	0.4	ND
	Tp	0.7	0.2		T_1_	0.7	2.1
**ID09**	T_0_	2.1	2.5		Tp	11.0	37.3
	T_1_	0.2	ND	**ID21**	T_0_	3.3	5.5
	T_2_	0.2	ND		T_1_	0.5	ND
	T_3_	0.3	ND		T_2_	0.4	0.8
	T_4_	0.1	ND		T_3_	0.3	1.0
	T_5_	0.4	1.4		T_4_	0.7	2.4
**ID10**	T_0_	1.8	7.3		Tp	2.5	7.6
	Tp	0.9	5.9	**ID22**	T_0_	0.8	0.7
**ID11**	T_0_	14.8	14.0		T_1_	0.8	ND
	T_1_	5.4	3.0		T_2_	0.4	ND
	Tp	7.1	5.5				

*Abbreviations*: ND (Not Detected); T_0_ (baseline timepoint); T_n_ (longitudinal timepoints); T_r_ (resistance timepoint) VAF (Variant allele fraction).

A median of 2.2 (ranging from 0.1 to 55.8) MI was calculated among all liquid biopsy samples. A median of 2.6 (ranging from 0.1 to 14.8) and 5.7 (ranging from 0.1 to 55.8) MI was identified at baseline and TP, respectively. SeqOne Genomics (Montpellier, France) successfully calculated MI in all instances overlapping with previous MI analysis (Supplementary Table 4). Beyond p. G12C hotspot mutation, no clinically actionable alterations were found in driver genes both at baseline and TP collection points. Filtering algorithm was designed as follows: synonymous, intronic and not assessed (NA) molecular alterations below 0.5% of VAF were discarded.

### 3.4 Early dynamic variations and patients’ outcomes

**Fig. 1:**
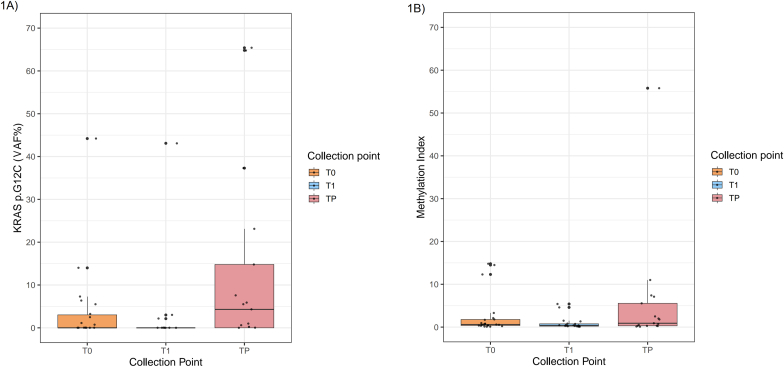
**(A)** Boxplot of *KRAS* p. G12C variant allelic fraction (VAF%) and **(B)** Methylation Index score at three timepoints: T0 (baseline), T1 (post-treatment), and TP (progression). Boxplots were created by using RStudio® (v.2025.05.1) showing the median, interquartile range (IQR) and individual outliers for each group.

Overall, genomic data from both baseline sample and first longitudinal timepoint (T1) were available in 19 out 22 (86.3%) NSCLC patients. Considering seven patients with a detectable *KRAS* p. G12C mutation at T0 and T1 samples, genomic analysis accurately revealed a decreasing mutation rate by comparing baseline and T1 in five cases (83.3%) (Δ = 2.6%, 0.7-5.5%); all of them achieved a complete ctDNA clearance of *KRAS* p. G12C mutation (Table 2, Fig. 1A) whereas a decreasing trend was identified in ID#02,11 (median Δ = 12.1%) (p-value = 0.07). Among the other n = 12 patients having undetectable p. G12C *KRAS* mutation at baseline, T1 positive signal was observed in a single instance (Table 2). Overall, methylome data both from baseline and T1 samples were available in all NSCLC patients. MI score was significantly lower (median 0.9, from 0.1 to 5.4; p-value = 0.04) in T1 samples compared with basal specimens (median 2.9, from 0.1 to 14.8) (Table 2, Fig. 1A).

In 13 out of 19 (68.4%) NSCLC patients MI decreased (4.1 vs 1.1 from Δ = 2.9, from 0.1 to 13.0). Conversely, 4 out of 19 (21.0%) NSCLC patients showed an increasing MI (0.2 vs 0.6; Δ = 0.5, from 0.2 to 1.1) in T1 samples, while two cases highlighted a stable MI (ID#08, ID#22) in comparison with baseline (Table 2).

**Fig. 2:**
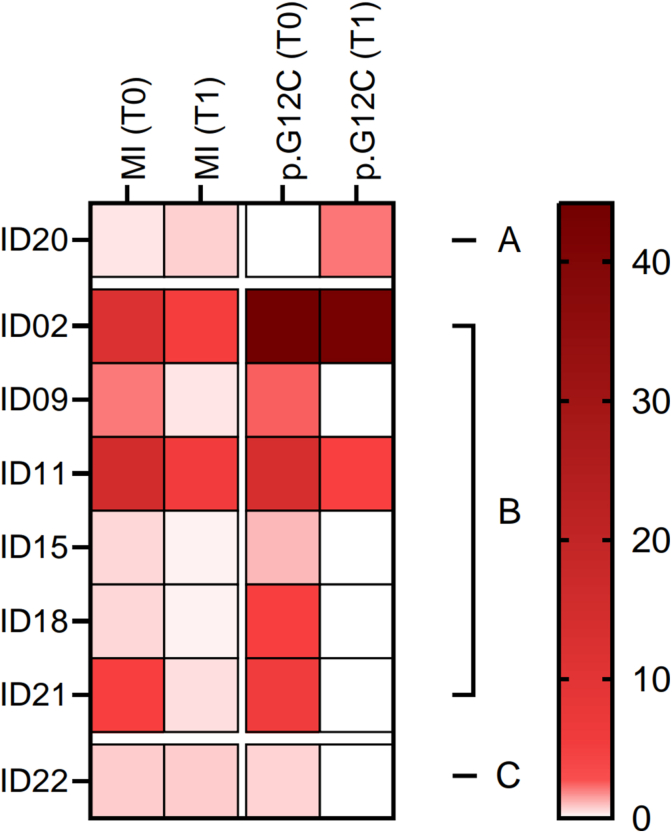
Heatmap with the variation of Methylation Index (MI) and *KRAS* p. G12C at T0 (baseline) and T1 (post-treatment). Each row represents a single patient; color intensity indicates the magnitude of calculating VAF and MI variations. Patients are clustered as follows: Group A (MI increase; p. G12C VAF increase); Group B (MI decrease; p. G12C VAF decrease); Group C (MI stable; p. G12C VAF decrease); in the graph T0-TP Group A (MI increase; p. G12C VAF increase); Group B (MI decrease; p. G12C VAF decrease); Group C (MI decrease; p. G12C VAF increase).

Matching p. G12C *KRAS* mutation and MI score, 1 out of 8 (12.5%) NSCLC patients experienced simultaneous improvement whereas a decreasing trend of p. G12C and MI was identified in 75.0% of cases (6 out of 8). Moreover, p. G12C *KRAS* mutation diminished concomitantly to a stable MI score (p.G12C VAF 0.7 to 0.0%, MI = 0.8) in a single case (12.5%) (Fig. 2).

### 3.5 Liquid biopsy data at sotorasib resistance

**Fig. 3:**
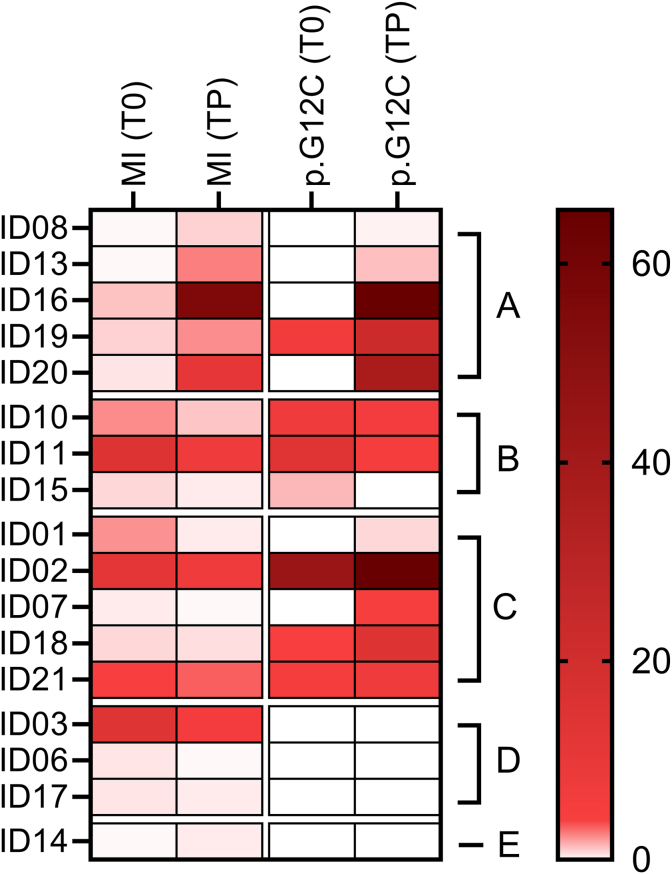
Heatmap with the variation of Methylation Index (MI) and *KRAS* p. G12C at and TP (progression). Each row represents a single patient; color intensity indicates the magnitude of calculating VAF and MI variations. Patients are clustered as follows: Group A (MI increase; p. G12C VAF increase); Group B (MI decrease; p. G12C VAF decrease); Group C (MI decrease; p. G12C VAF increase).

A total of 17 out of 22 (77.3%) patients with liquid biopsy samples available within 90 days of radiologic progression disease (PD) were selected. Of note, 12 out of 17 (70.6%) patients highlighted positive signal (VAF≥ 0.2%) of *KRAS* p. G12C at TP. Not surprisingly, a median p. G12C VAF of 19.2% (from 0.2 to 65.4%) was observed in TP samples. (median 11.7%, range 1.1- 44.2%) (p-value = 0.06) (Table 2, Fig. 1B). Particularly, in 10 out of 17 (58.8%) cases, median VAF of p. G12C increased (5.9%, 0.0 to 44.2% at baseline vs 21.9%, 0.2 to 65.4% at TP) (Δ = 16.0%, from 0.2% to 64.8%) (Table 2, Fig. 1B). Conversely, three patients (17.6%) highlighted a decreasing rate of p. G12C (median VAF 7.5%, 1.1 to 14.0% at baseline vs 3.8%, 0.0 to 5.9% at TP) (Δ = 3.6%, from 1.1% to 8.5%). Remaining cases revealed stable p. G12C values between T0 and TP**.** (Table 2). MI was higher in TP (median 5.7, from 0.1 to 55.8) compared with baseline samples (median 3.1, from 0.1 to 14.8 p value = 0.46) (Fig. 1B). In addition, an increasing trend between baseline and TP (0.4 vs 12.0; Δ = 11.5, from 0.2 to 54.9) was identified in 6 out of 17 (35.3%) NSCLC patients (Table 2). Interestingly, 5 out of 13 (38.5%) NSCLC patients displayed a simultaneously increasing rate of *KRAS* p. G12C mutation and MI score whereas in 23.1% of cases (3 out of 13) both p. G12C *KRAS* mutation and MI decreased. In addition, a divergent trend, where p. G12C *KRAS* raised with progressive decrease of MI at TP, was identified in 5 out of 13 NSCLC patients (38.5%) (Fig. 3).

### 3.6 Methylation index and patients’ outcomes

**Fig. 4:**
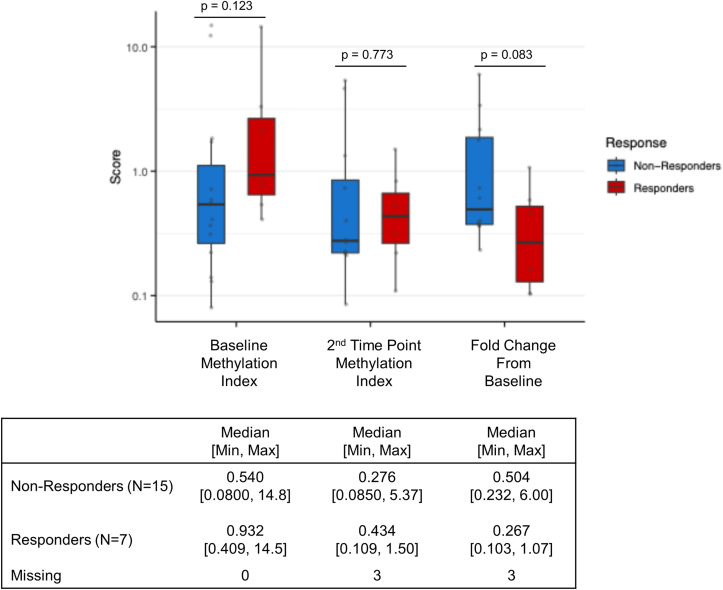
Correlation between Methylation Index (MI) and radiological response to sotorasib across different timepoints.

**Supplementary Fig. 3:**
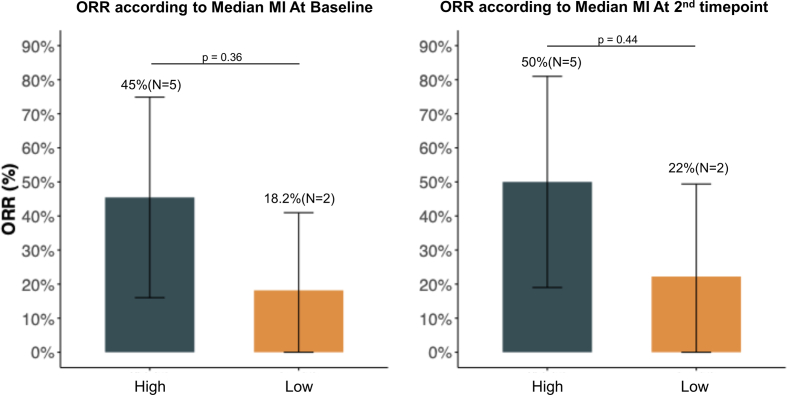
Objective Response Rate to sotorasib therapy according to the median Methylation Index (MI) across different timepoints.

**Supplementary Fig. 4A, B:**
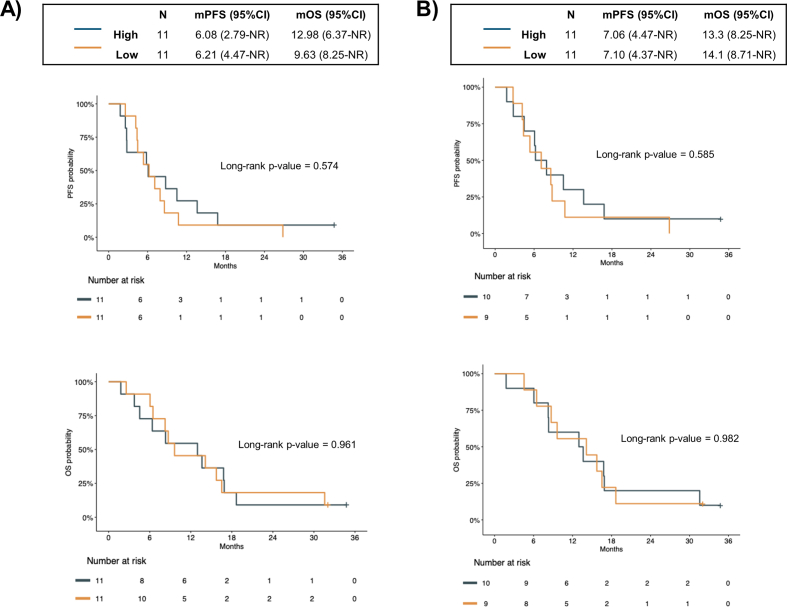
Median progression free survival (mPFS) and median overall survival (mOS) of KRASp.G12C mutated advanced NSCLC patients undergoing sotorasib therapy according to the median Methylation Index (MI) at baseline (A) and at the first collection timepoints (B).

Among the n = 22 NSCLC patients, any potential differences of baseline median MI across the main patients’ subgroups were explored and the results were summarized in Table 1. A trend toward an increased median MI was found in patients with PD-L1 positive vs negative tumors (3.30 vs 0.55, p:0.164) but statistically significant variation was not assessed. Considering n = 19 NSCLC patients with T1 collecting points, a not significant trend toward both an increased median MI (0.43 vs 0.27, p: 0.773) and a decreased MI fold-change (0.267 vs 0.504, p:0.08) was found in responders (PR) vs non-responders (SD + PD) (Fig. 4). Setting the median MI as reference cut-off, an increased ORR was observed in patients with high vs low MI detected both at baseline (45.5% vs 18.2%, p:0.36) and at T1 (50.0% vs 22.2%, p: 0.44) timepoints, even if variations did not reach statistical significance (Supplementary Figure 3). Similar median PFS and OS emerged in NSCLC patients with high MI compared with paired baseline samples (mPFS: 6.08 vs 6.21 months, p: 0.57; mOS: 13 vs 9.6 months, p:0.96) (Supplementary Figure 4A) and at T1 (mPFS: 7.06 vs 7.10 months, p: 0.58; mOS: 13.3 vs 14.1 months, p:0.98) timepoints (Supplementary Figure 4B). Setting the median MI fold change between baseline and T1 as reference cut-off, no differences in terms of ORR (40% vs 33%, p:1) and mPFS (7.43 vs 7.10, p: 0.81) (Supplementary Fig. 5A–B) were observed, while a not significant trend toward increased mOS (15.3 vs 9.63, p: 0.77) (Supplementary Figure 5C) were identified in patients with low vs high MI fold-change values.

## 4 Discussion

In the era of personalized treatments, an accurate stratification of NSCLC patients is pivotal. Given the rapidly transforming scenario of clinically approved biomarkers, including genomic alterations and aberrant RNA rearrangements, NSCLC patients may benefit from several drugs selectively targeting specific oncoproteins. Despite these advances, a consistent fraction of NSCLC patients with driver molecular alterations experienced a limited clinical benefit from current targeted therapies. In this scenario, epigenomic markers (chromatin remodeling, methylation status, histone modifications) may impact on the clinical selection of NSCLC patients guiding clinical decision-making procedures. Here, we sought to evaluate how methylation index, calculated by NGS panel integrating genomic and methylome analysis, may optimize clinical stratification of p. G12C *KRAS* positive NSCLC patients undergoing sotorasib treatment. A series of n = 22 NSCLC patients were investigated by multilayer NGS panel (Avida Duo Methyl Reagent Kit, Avida Biomed) automatically scoring MI by proprietary bioinformatic pipeline able to assess methylation patterns of 3400 cancer-related CpGs after bisulfite conversion. Comparing bisulfite-based methods with other strategies for DNA methylation profiling, lower technical performance in terms of reference range (>28 million of CpGs vs < 23 million of CpGs covered) and technical resolution (comprehensive CpGs analysis vs target CpGs patterns) were observed in affinity enrichment and restriction enzymes-based methods [20]. Dynamic evaluation of ctDNA was inspected at T1 and TP simultaneously investigating p. G12C *KRAS* driver mutation and MI score. Before molecular analysis, cfDNA abundance was calculated as 170/700 bp ratio by microfluidic system showing that no statistically significant variations were observed between baseline, TI and TP samples (72.0, 72.0, 77.0 cfDNA%, respectively, p-value = 0.21).

Interestingly, 6 out of 7 (85.7%) patients showing traces of p. G12C alteration at baseline decreasing p. G12C level at T1 and achieving a complete clearance of in 83.3% of cases (Table 2). Switching to progression, p. G12C *KRAS* mutation was found in 70.6% of cases demonstrating a significant variation of the median VAF comparing baseline and TP (11.7 % vs 19.2%) (p-value = 0.07) (Fig. 1A). In 10 out of 13 (76.9%) NSCLC patients, p. G12C *KRAS* increased at TP (5.9% vs 21.9% VAF). In line with previous data, persisting ctDNA traces in longitudinal time points correlated with worse clinical outcomes. Moreover, abundance of ctDNA depending on higher p. G12C VAF at TP can also impact on the clinical response and relapsing timeline. In a previous metanalysis, Zaman et al. highlighted that ctDNA-negative patients at baseline had a longer PFS (pooled hazard ratio [pHR] = 1.35; 95%CI: 0.83–1.87; *p* < 0.001; I^2^ = 96%) compared with baseline ctDNA positive. In addition, clearance of ctDNA levels after target treatment was significantly associated with increasing PFS (pHR = 2.71; 95%CI: 1.85–3.65; I^2^ = 89.4%) in comparison with persistence ctDNA levels at recollection points [21]. Similarly, Passiglia et al. also confirmed that early ctDNA clearance detecting p. G12C *KRAS* mutation correlated with longer OS (16.8 vs. 6.4 months; p < 0.001) in advanced NSCLC patients under sotorasib, while increasing of VAF anticipated radiological PD, suggesting a potential role for ctDNA driven escalation and de-escalation strategies in *KRAS* mutated patients [22].

**Fig. 5:**
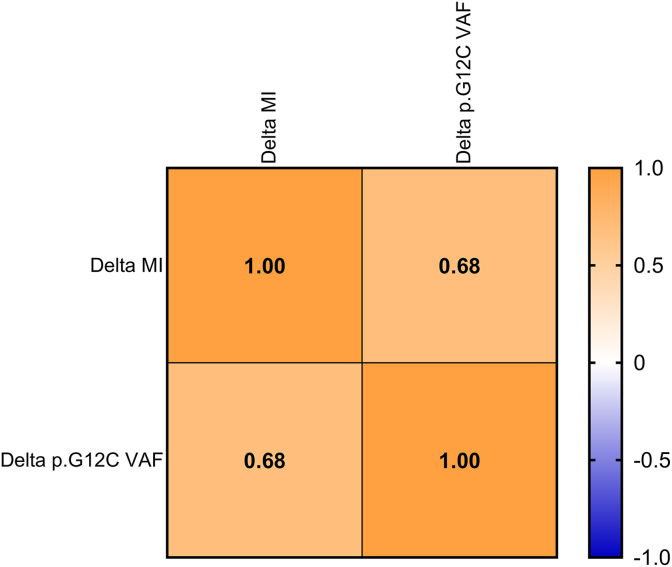
Correlation matrix between changes in Methylation index (Delta MI) and KRAS p. G12C VAF among different times points (T0/T1). The heatmap displays Pearson correlation coefficients between Delta MI and Delta p. G12C hot spot mutation. The color gradient represents the magnitude and direction of the correlation, ranging from blue (negative correlation) to orange (positive correlation). A moderate positive correlation (r = 0.68, *p* = 0.06) was observed between Delta MI and Delta p. G12C alteration, suggesting a positive association between T0 and T1, although not statistically significant association setting threshold at *p* < 0.05 was achieved.

**Fig. 6:**
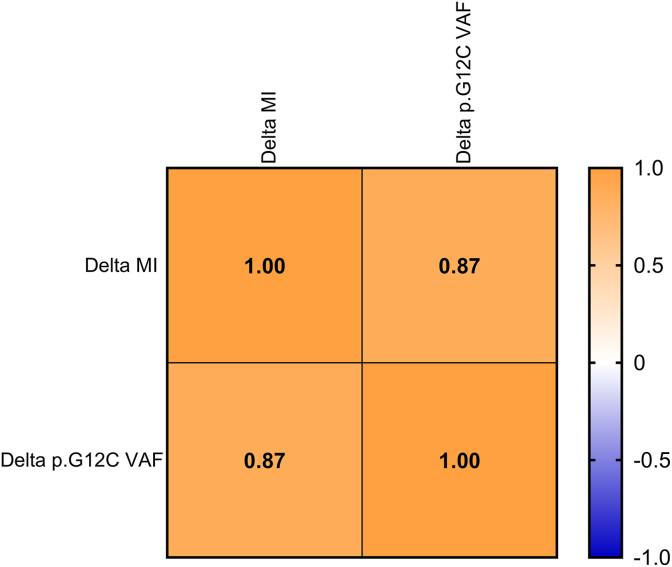
Correlation matrix between changes in Methylation index (Delta MI) and KRAS p. G12C VAF among different times points (T0/TP). The heatmap displays Pearson correlation coefficients between Delta MI and Delta p. G12C hot spot mutation. The color gradient represents the magnitude and direction of the correlation, ranging from blue (negative correlation) to orange (positive correlation). A strong positive correlation was observed between Delta MI and Delta p. G12C alteration (r = 0.87, p = 0.000103), indicating a statistically significant association between Methylation changes and p. G12C abundance from T0 to TP.

**Supplementary Fig. 6:**
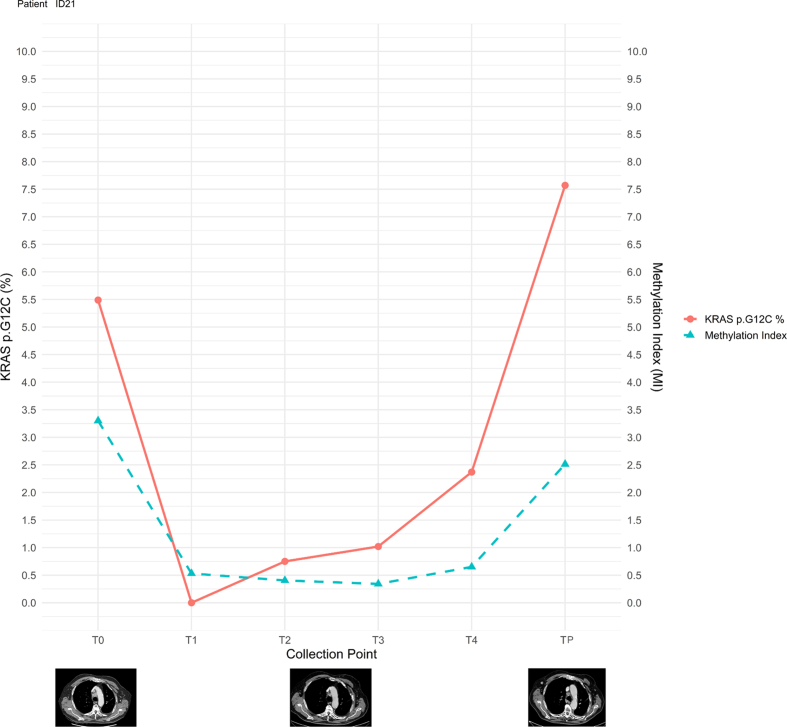
Longitudinal variations of p.G12C *KRAS* mutation and MI score across collection points of patient ID#21.

The clinical evidence confirms that the clinical response of p. G12C mutant NSCLC patients treated with target therapy is modulated by the clearance of driver alteration. Paweletz et al. highlighted that p. G12C clearance within cycle 2 had a higher objective response rate (ORR) compared with persistent p. G12C series (60.6% vs 33.3%) [23]. Conversely, the lack of common driver mechanisms between basal and longitudinal samples (baseline vs recollection; baseline vs TP) suggested that additional resistance mechanisms should be explored beyond genetic signatures. [24,25]. On this basis, methylation pattern may significantly impact on the clinical outcomes of NSCLC patients driven by *KRAS* mutations. As shown, MI correlates with a better clinical outcome at baseline paving the way for tailored treatments targeting neoplastic cells. Interestingly, higher MI score at progression can significantly track clinical progression faster predicting recurrence than conventional techniques [[26], [27], [28]]. We successfully calculated MI in each sample by adopting both proprietary bioinformatic pipeline and SeqOne analysis software (Montpellier, France) (Supplementary Table 4). The heterogeneous landscape of analytical strategies measuring methylation profile significantly affects the clinical application of methylome signature [29,30]. No statistically significant variations (p value = 0.9) between proprietary bioinformatic pipeline and SeqOne analysis software (Montpellier, France) calculating MI from methylome analysis paving the way for the clinical applications of these tools. In particular, 68.4% of NSCLC patients highlighted a consistent decreasing rate of MI (4.1 vs 1.1 from Δ = 2.9, from 0.1 to 13.0) at T1, in line with *KRAS* p. G12C mutation clearance (57.1%) (p-value = 0.04) (Fig. 2, Fig. 5). Of note, MI significantly arised in 35.3% (6 out of 17) of NSCLC patients at TP compared with baseline samples (12.0 vs 0.4; Δ = 11.5, from 0.2 to 54.9) matching with *KRAS* p. G12C mutation increase level (Fig. 3, Fig. 6). Remarkably, MI highlighted a similar trend of *KRAS* p. G12C VAF among baseline, TI and TP points (median VAF 0.0, 0.0, 2.1% - median MI 0.6, 0.3, 0.9, respectively, Person correlation basal and T1 = 0.68; Person correlation basal and TP = 0.87) (Fig. 5, Fig. 6) Noteworthy, further investigations are needed to consolidate positive trends correlating genomic and methylome data. Even if limited by small sample size, this data suggests that dynamic modification of both methylation patterns and genomic assessment may track tumor under KRASG12C inhibitors. This proof of concept is clear by looking at two patients of the clinical series, with five samples collecting points availability, demonstrating significantly correlated longitudinal variations of genomic and/or methylome data. ID#16 showed a significant peak both in *KRAS* p. G12C VAF and MI score at TP compared with other longitudinal timepoints (from 0.0 to 64.8%; 0.9 to 55.8, respectively) (Table 2). Interestingly, ID#21 was affected by simultaneous variations of p. G12C and MI score dynamically modified among the different collection timepoints. Moving from T0 to T1, *KRAS* p. G12C was undetectable while a positive MI score was identified predicting clinical recurrence. As shown, MI score can consistently modify clinical management of NSCLC patients integrating genomic profile in longitudinal monitoring of tumor evolution [12]. (Supplementary Fig. 6) Despite the powerful insights, several limitations should be considered. Firstly, sample set was retrospectively retrieved without any statistical tool calculating sample size. Moreover, scant number of n = 22 retrospective NSCLC patients can affect statistical analysis impacting on clinical correlation of molecular data. Secondly, MI was calculated on 3400 differentially methylated regions (DMRs) partially evaluating methylome signature in comparison with whole genome approaches. Thirdly, MI cutoff was set on prespecified small cohort of patients partially capturing epigenetic profile of validation series of NSCLC patients. Finally, further investigations are required to validate clinical role of methylation profile in the clinical management of *KRAS* mutated NSCLC patients comprehensively investigating co-occurring genomic hallmarks that could impact on dynamic evolution of clinical outcomes. In conclusion, methylation signature integrating genomic analysis may represent an informative tool successfully optimizing personalized therapeutic strategies for *KRAS* p. G12C mutant NSCLC patients.

## Ethics approval statement

All information regarding human material was managed using anonymous numerical codes, and all samples were handled in compliance with the Helsinki Declaration. The PROMOLE protocol was previously approved by the Independent Ethic Committee of S. Luigi Hospital, University of Turin (ethics approval number 73/2018 of 2024.01.30).

## Funding

This study has partly been supported by the following grants: 1. “NExt generation Omics-NEON” bando a cascata Anthem - prot. n. 0001983 - CUP B53C22006700001. 2. The Italian Health Ministry's Research Program (ID: NET-2016–02363853). 3. The National Center for Gene Therapy and Drugs based on RNA Technology MUR-CN3 CUP E63C22000940007 to DS. 4. Italian Ministry of Health (Piano Operativo Salute Traiettoria 3, T3-AN-09, “Genomed”. 5. PRIN-Project ID: P2022L4CK4, funding code 000018 PRIN_PNRR_2022, CUP E53D23015450001. 6. HERA-ORION-D3 4 Health, PNC – CUP B53C22005980001, PNC0000001_UNINA_HERA_ORION.

## Declaration of competing interest

The authors declare the following financial interests/personal relationships which may be considered as potential competing interests: Francesco Pepe has received personal fees (as consultant and/or speaker bureau) from Menarini, Roche, Thermofisher, Jansen unrelated to the current work; Francesco Passiglia received speakers' and consultants' fee from AstraZeneca, Johnson&Johnson, Novartis, Roche, MSD, Amgen, Beone, Gilead, Pharmamar, Thermo Fisher Scientific unrelated to the current work. Luisella Righi received speakers' and consultants’ fee from AstraZeneca, Novartis, Roche, Amgen, BeiGene, Novartis, EliLilly un related to the current work. Paul Hofman received fee and honoraria from AstraZeneca Roche Amgen Biocartis Thermo Fisher Scientific BMS MSD abbvie pierre Fabre Pfizer Novartis Daiichi Sankyo Ed Lilly Biodena Merck unrelated to the current work. Silvia Novello reports personal fees (as speaker bureau or advisor) from Eli Lilly, MSD, Roche, Takeda, Pfizer, Astra Zeneca, Amgen, Thermo Fisher, Novartis, Sanofi, Johnson&Johnson outside the current work. Giancarlo Troncone reports personal fees (as speaker bureau or advisor) from Roche, MSD, Pfizer and Bayer, unrelated to the current work; Umberto Malapelle has received personal fees (as consultant and/or speaker bureau) from Boehringer Ingelheim, Roche, MSD, Amgen, Thermo Fisher Scientific, Eli Lilly, Diaceutics, GSK, Merck and AstraZeneca, Janssen, Diatech, Novartis and Hedera for work performed. The remaining authors declare that they have no known competing financial interests or personal relationships that could have appeared to influence the work reported in this paper.

## Figures and Tables

**Supplementary Fig. . 5A-C:**
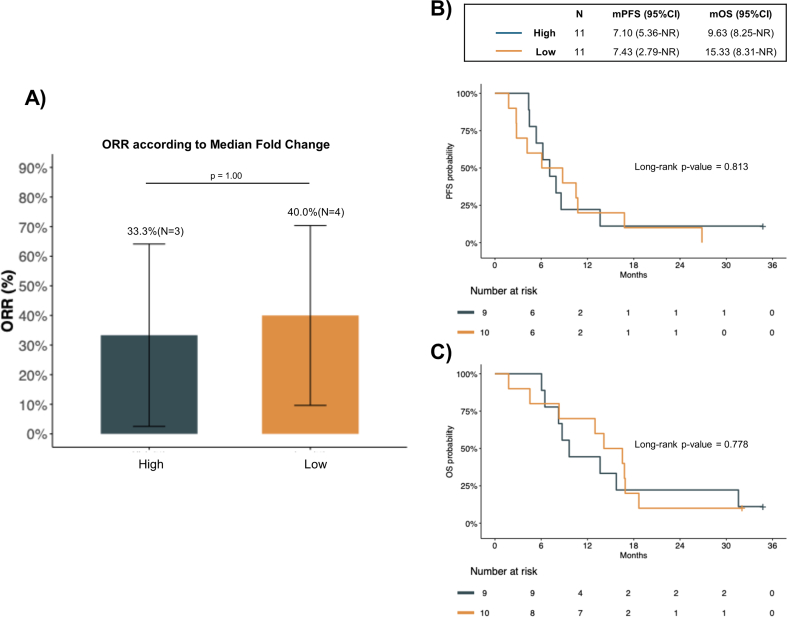
Objective Response Rate (ORR) (A), Median progression free survival (mPFS) (B) and median overall survival (mOS) (C) of KRASp.G12C mutated advanced NSCLC patients undergoing sotorasib therapy according to the median fold change of Methylation Index (MI) between baseline and at the first collection timepoints.
