# Supplementary material for: Exploring genomic analysis and methylome profiling in longitudinal series of p.G12C KRAS mutated NSCLC patients treated with sotorasib
Source: J Liq Biopsy. 2026 Apr 27;12:100467. doi: 10.1016/j.jlb.2026.100467 (PMC13146550; doi:10.1016/j.jlb.2026.100467)
Supplement: Multimedia component 1 [file mmc1.docx]

| **ID sample** | **Collection point** | **cfDNA concentration (ng/μl)** | **% cfDNA** |  | **ID sample** | **Collection point** | **cfDNA concentration (ng/μl)** | **% cfDNA** |
| --- | --- | --- | --- | --- | --- | --- | --- | --- |
| **ID01** | T_0_ | 0.1 | 78.0% |  | **ID12** | T_0_ | 0.1 | NA |
|  | T_1_ | 0.1 | 74.0% |  |  | T_1_ | 0.1 | 64.0% |
|  | T^r^ | 0.1 | 75.0% |  |  | T_2_ | 0.1 | NA |
| **ID02** | T_0_ | 0.3 | 81.0% |  |  | T_3_ | 0.1 | 70.0% |
|  | T_1_ | 0.1 | 79.0% |  |  | T_4_ | 0.1 | 67.0% |
|  | T^r^ | 0.2 | 87.0% |  |  | T_5_ | 0.1 | 67.0% |
| **ID03** | T_0_ | 0.1 | 79.0% |  |  | T_6_ | 0.1 | 64.0% |
|  | T_1_ | 0.1 | NA |  | **ID13** | T_0_ | 0.1 | 56.0% |
|  | T_2_ | 0.1 | NA |  |  | T_1_ | 0.3 | 64.0% |
|  | T^r^ | NA | NA |  |  | T_2_ | 0.4 | 76.0% |
| **ID04** | T_0_ | 0.1 | NA |  |  | T_3_ | 0.4 | 74.0% |
|  | T_1_ | 0.2 | 81.0% |  |  | T_4_ | 0.2 | 55.0% |
| **ID05** | T_0_ | 0.1 | 76.0% |  |  | T_5_ | 0.3 | 57.0% |
|  | T_1_ | 0.1 | 74.0% |  |  | T^r^ | 0.2 | 75.0% |
|  | T_2_ | 0.1 | 73.0% |  | **ID14** | T_0_ | 0.2 | 66.0% |
|  | T_3_ | 0.1 | 74.0% |  |  | T_1_ | 0.2 | 52.0% |
|  | T_4_ | 0.1 | 81.0% |  |  | T^r^ | 0.3 | 57.0% |
|  | T_5_ | 0.1 | 71.0% |  | **ID15** | T_0_ | 0.2 | 62.0% |
|  | T_6_ | 0.1 | 82.0% |  |  | T_1_ | 0.1 | NA |
|  | T_7_ | 0.1 | 77.0% |  |  | T_2_ | 0.1 | 81.0% |
| **ID06** | T_0_ | 0.1 | 81.0% |  |  | T^r^ | 0.1 | 62.0% |
|  | T_1_ | 0.1 | 71.0% |  | **ID16** | T_0_ | 0,2 | 83.0% |
|  | T_2_ | 0.1 | 78.0% |  |  | T_1_ | 0.1 | 79.0% |
|  | T^r^ | 0.1 | 83.0% |  |  | T_2_ | 0.1 | 74.0% |
| **ID07** | T_0_ | 0.1 | NA |  |  | T_3_ | 0.1 | 76.0% |
|  | T_1_ | 0.1 | 66.0% |  |  | T^r^ | 0.3 | 83.0% |
|  | T_2_ | 0.1 | 73.0% |  | **ID17** | T_0_ | 0.8 | 83.0% |
|  | T^r^ | 0.2 | 77.0% |  |  | T^r^ | 0.4 | 78.0% |
| **ID08** | T_0_ | 0.1 | 67.0% |  | **ID18** | T_0_ | 0.2 | 80.0% |
|  | T_1_ | 0.9 | 91.0% |  |  | T_1_ | 0.2 | 78.0% |
|  | T_2_ | 1.3 | 92.0% |  |  | T^r^ | 0.2 | 74.0% |
|  | T_3_ | 2.4 | 93.0% |  | **ID19** | T_0_ | 0.1 | 54.0% |
|  | T_4_ | 0.8 | 92.0% |  |  | T^r^ | 0.3 | 74.0% |
|  | T_5_ | 1.3 | 93.0% |  | **ID20** | T_0_ | 0.1 | 65.0% |
|  | T^r^ | 1.3 | 91.0% |  |  | T_1_ | NA | NA |
| **ID09** | T_0_ | 0.2 | 73.0% |  |  | T^r^ | 0.1 | 73.0% |
|  | T_1_ | 0.1 | 68.0% |  | **ID21** | T_0_ | 0.2 | 76.0% |
|  | T_2_ | 0.1 | 73.0% |  |  | T_1_ | 0.1 | 64.0% |
|  | T_3_ | 0.2 | 32.0% |  |  | T_2_ | 0.2 | 64.0% |
|  | T_4_ | NA | NA |  |  | T_3_ | 0.2 | 71.0% |
|  | T_5_ | 0.1 | NA |  |  | T_4_ | 0.3 | 77.0% |
| **ID10** | T_0_ | 0.1 | NA |  |  | T^r^ | 0.3 | 80.0% |
|  | T^r^ | 0.1 | NA |  | **ID22** | T_0_ | 0.2 | 53.0% |
| **ID11** | T_0_ | 0.1 | 73.0% |  |  | T_1_ | 0.2 | 72.0% |
|  | T_1_ | 0.2 | 69.0% |  |  | T_2_ | 0.1 | 62.0% |
|  | T^r^ | 0.3 | 81.0% |  |  |  |  |  |

**Supplementary table 1***:* CfDNA abundance measured by microfluidic system in longitudinal series of plasma samples.

*Abbreviations*: cfDNA (cell-free DNA); T_0_ (Baseline timepoint); T_1_ (first longitudinal timepoint); T_r_ (resistance timepoint)
