# Supplementary material for: Exploring genomic analysis and methylome profiling in longitudinal series of p.G12C KRAS mutated NSCLC patients treated with sotorasib
Source: J Liq Biopsy. 2026 Apr 27;12:100467. doi: 10.1016/j.jlb.2026.100467 (PMC13146550; doi:10.1016/j.jlb.2026.100467)
Supplement: Multimedia component 2 [file mmc2.docx]

| **ID Sample** | **Collection point** | **Total number of raw reads** | **Number of mapped reads** | **Number of high-quality reads** | **Median depth in targeted region** |
| --- | --- | --- | --- | --- | --- |
| **ID01** | T_0_ | 9380908 | 1525084 | 1423393 | 172 |
|  | T_1_ | 9745436 | 1832938 | 1748637 | 243 |
|  | T_r_ | 7895136 | 1332370 | 1297583 | 210 |
| **ID02** | T_0_ | 7305996 | 3316830 | 3273103 | 549 |
|  | T_1_ | 9136986 | 2172728 | 2116549 | 332 |
|  | T_r_ | 9981162 | 1599914 | 1533693 | 223 |
| **ID03** | T_0_ | 8729100 | 815362 | 785281 | 122 |
|  | T_1_ | 8204746 | 765246 | 739589 | 115 |
|  | T_2_ | 6327536 | 737234 | 718769 | 118 |
|  | T_r_ | 5139660 | 886650 | 863330 | 135 |
| **ID04** | T_0_ | 6275666 | 802940 | 780903 | 126 |
|  | T_1_ | 8571384 | 2992154 | 2948473 | 486 |
| **ID05** | T_0_ | 6753412 | 1141088 | 1113542 | 182 |
|  | T_1_ | 7794518 | 1433102 | 1397525 | 225 |
|  | T_2_ | 7459064 | 1687288 | 1648492 | 263 |
|  | T_3_ | 6910602 | 1032110 | 1004115 | 160 |
|  | T_4_ | 7345956 | 1402248 | 1371861 | 224 |
|  | T_5_ | 8702130 | 1884982 | 1836371 | 290 |
|  | T_6_ | 9371346 | 2303780 | 2247036 | 358 |
|  | T_7_ | 9663024 | 2262366 | 2221020 | 365 |
| **ID06** | T_0_ | 7511288 | 1421108 | 1381793 | 213 |
|  | T_1_ | 8275620 | 1654758 | 1617968 | 260 |
|  | T_2_ | 8572920 | 1677970 | 1640935 | 267 |
|  | T_r_ | 9302052 | 2534394 | 2482427 | 392 |
| **ID07** | T_0_ | 9284952 | 725400 | 669356 | 96 |
|  | T_1_ | 7740710 | 1241048 | 1195744 | 179 |
|  | T_2_ | 10126710 | 1957216 | 1889014 | 277 |
|  | T_r_ | 8622956 | 2914726 | 2851463 | 444 |
| **ID08** | T_0_ | 11614104 | 7148178 | 7037096 | 1062 |
|  | T_1_ | 12005772 | 8127454 | 8002594 | 1171 |
|  | T_2_ | 11206256 | 8178296 | 8067187 | 1237 |
|  | T_3_ | 11289336 | 9473110 | 9345284 | 1392 |
|  | T_4_ | 11433068 | 8993788 | 8887444 | 1397 |
|  | T_5_ | 11064974 | 8832400 | 8707268 | 1298 |
|  | T_r_ | 11930904 | 9415236 | 9302484 | 1500 |
| **ID09** | T_0_ | 9993382 | 2653828 | 2591402 | 399 |
|  | T_1_ | 9807440 | 3375324 | 3217150 | 400 |
|  | T_2_ | 10486330 | 3466630 | 3352785 | 473 |
|  | T_3_ | 11308968 | 3183428 | 3098317 | 469 |
|  | T_4_ | 9593054 | 1187424 | 1124248 | 164 |
|  | T_5_ | 8960878 | 1746370 | 1675990 | 238 |
| **ID10** | T_0_ | 9565498 | 3845200 | 3727869 | 515 |
|  | T_r_ | 9592808 | 3381078 | 3264757 | 450 |
| **ID11** | T_0_ | 8525608 | 1818118 | 1729583 | 245 |
|  | T_1_ | 7609090 | 2126178 | 2021963 | 267 |
|  | T_r_ | 8413010 | 2428534 | 2376123 | 368 |
| **ID12** | T_0_ | 9658622 | 458720 | 424865 | 63 |
|  | T_1_ | 10029914 | 1472094 | 1403367 | 208 |
|  | T_2_ | 9175098 | 1417892 | 1366169 | 202 |
|  | T_3_ | 10308748 | 2191366 | 2137273 | 336 |
|  | T_4_ | 11299212 | 2403146 | 2330046 | 343 |
|  | T_5_ | 11756218 | 2261052 | 2193187 | 333 |
|  | T_6_ | 13194652 | 3891026 | 3807463 | 566 |
| **ID13** | T_0_ | 9560712 | 1283180 | 1222831 | 176 |
|  | T_1_ | 10824162 | 1824988 | 1746353 | 248 |
|  | T_2_ | 12542894 | 3536652 | 3442660 | 512 |
|  | T_3_ | 17458400 | 6371936 | 6149817 | 838 |
|  | T_4_ | 12122368 | 2941052 | 2821749 | 385 |
|  | T_5_ | 11923242 | 2640056 | 2539095 | 364 |
|  | T_r_ | 16748636 | 4011508 | 3902588 | 589 |
| **ID14** | T_0_ | 12932308 | 2949110 | 2881517 | 461 |
|  | T_1_ | 14841514 | 2455560 | 2395122 | 388 |
|  | T_r_ | 10928480 | 2142434 | 2097016 | 364 |
| **ID15** | T_0_ | 11264942 | 1102790 | 1053551 | 164 |
|  | T_1_ | 10172702 | 1397702 | 1331126 | 199 |
|  | T_2_ | 10769888 | 1522446 | 1456627 | 221 |
|  | T_r_ | 10455554 | 917728 | 867200 | 136 |
| **ID16** | T_0_ | 13105402 | 3005336 | 2924654 | 458 |
|  | T_1_ | 8056940 | 1785536 | 1730390 | 267 |
|  | T_2_ | 11599032 | 1788170 | 1723767 | 264 |
|  | T_3_ | 11663760 | 1513164 | 1454410 | 227 |
|  | T_r_ | 12672958 | 3389248 | 3312445 | 524 |
| **ID17** | T_0_ | 17276150 | 11360524 | 11077138 | 1617 |
|  | T_r_ | 13634070 | 6565818 | 6368157 | 932 |
| **ID18** | T_0_ | 24730810 | 3696514 | 3452583 | 478 |
|  | T_1_ | 28249470 | 5699460 | 5387986 | 743 |
|  | T_r_ | 20416722 | 3779948 | 3562548 | 489 |
| **ID19** | T_0_ | 18516046 | 1818588 | 1683918 | 233 |
|  | T_r_ | 45165008 | 8672726 | 8325314 | 1191 |
| **ID20** | T_0_ | 17324840 | 1827332 | 1691911 | 236 |
|  | T_1_ | 19629550 | 1909020 | 1757363 | 232 |
|  | T_r_ | 25087696 | 3659344 | 3434843 | 462 |
| **ID21** | T_0_ | 22913444 | 4583004 | 4347456 | 599 |
|  | T_1_ | 21382416 | 2486998 | 2269520 | 297 |
|  | T_2_ | 21813862 | 2955534 | 2713567 | 349 |
|  | T_3_ | 18979324 | 3837656 | 3636523 | 497 |
|  | T_4_ | 22170908 | 5426786 | 5110287 | 636 |
|  | T_r_ | 23181062 | 6076836 | 5693899 | 659 |
| **ID22** | T_0_ | 14000856 | 1811772 | 1723212 | 264 |
|  | T_1_ | 23066318 | 3854528 | 3671389 | 550 |
|  | T_2_ | 16738364 | 1648978 | 1485475 | 187 |

**Supplementary table 2A**: List of technical parameters supporting *KRAS* p.G12C genomic analysis by proprietary bioinformatic pipeline of Avida Duo Methyl Reagent Kit (Avida Biomed).

*Abbreviations*: T_0_ (Baseline timepoint); T_1_ (first longitudinal timepoint); T_r_ (resistance timepoint)
