# Supplementary material for: Exploring genomic analysis and methylome profiling in longitudinal series of p.G12C KRAS mutated NSCLC patients treated with sotorasib
Source: J Liq Biopsy. 2026 Apr 27;12:100467. doi: 10.1016/j.jlb.2026.100467 (PMC13146550; doi:10.1016/j.jlb.2026.100467)
Supplement: Multimedia component 3 [file mmc3.docx]

| **ID Sample** | **Collection point** | **Total number of raw reads** | **Number of mapped reads** | **Number of high quality reads** | **Total number of analyzed cytosines** | **Methylated C in CpG context** | **Methylated C in CHG context** | **Methylated C in CHH context** |
| --- | --- | --- | --- | --- | --- | --- | --- | --- |
| **ID01** | T_0_ | 29719344 | 6374726 | 6080322 | 3366721 | 7.5 | 1.8 | 1.9 |
|  | T_1_ | 27293502 | 6910682 | 6499660 | 4507789 | 8.5 | 3.7 | 4.3 |
|  | T_r_ | 29076372 | 6302170 | 5896486 | 2698489 | 9.9 | 3.7 | 3.8 |
| **ID02** | T_0_ | 29130702 | 5499154 | 5142426 | 9631078 | 14.7 | 2.8 | 2.7 |
|  | T_1_ | 25305612 | 6673808 | 6216562 | 3301847 | 12.8 | 4.0 | 4.1 |
|  | Tr | 29275870 | 6450528 | 6042562 | 3063522 | 16.3 | 4.3 | 4.3 |
| **ID03** | T_0_ | 33099342 | 5370724 | 4872152 | 651065 | 17.8 | 4.3 | 5.0 |
|  | T_1_ | 47794630 | 6330876 | 5896074 | 1415255 | 14.9 | 7.7 | 7.6 |
|  | T_2_ | 14433052 | 7053392 | 6721574 | 1460530 | 7.5 | 1.2 | 1.1 |
|  | Tr | 3746190 | 2423128 | 2228134 | 858116 | 10.1 | 1.6 | 1.7 |
| **ID04** | T_0_ | 22435404 | 6433286 | 6019352 | 1660564 | 7.4 | 1.3 | 1.3 |
|  | T_1_ | 20928424 | 6314656 | 5940956 | 8080908 | 10.7 | 1.4 | 1.4 |
| **ID05** | T_0_ | 21465210 | 6262440 | 5864456 | 1989232 | 7.9 | 2.8 | 3.3 |
|  | T_1_ | 19228656 | 6879208 | 6445410 | 4396158 | 8.2 | 2.2 | 2.2 |
|  | T_2_ | 25434314 | 6858512 | 6400362 | 4798155 | 8.1 | 2.6 | 2.5 |
|  | T_3_ | 19267630 | 6833320 | 6365978 | 2389019 | 10.9 | 4.5 | 4.5 |
|  | T_4_ | 12486926 | 6888336 | 6487212 | 5024456 | 5.7 | 0.6 | 0.6 |
|  | T_5_ | 25046456 | 6858692 | 6419890 | 4871505 | 5.6 | 0.5 | 0.5 |
|  | T_6_ | 26195046 | 6804056 | 6388434 | 5246252 | 5.7 | 0.5 | 0.5 |
|  | T_7_ | 21717328 | 6846284 | 6345714 | 4818952 | 6.1 | 0.7 | 0.7 |
| **ID06** | T_0_ | 20837254 | 6977340 | 6565902 | 3565582 | 5.3 | 0.6 | 0.6 |
|  | T_1_ | 14974866 | 7031026 | 6571394 | 4490005 | 5.3 | 0.7 | 0.7 |
|  | T_2_ | 22733516 | 7044138 | 6637426 | 4356224 | 5.5 | 0.7 | 0.7 |
|  | T_r_ | 8201314 | 2829510 | 2565002 | 3903419 | 6.7 | 1.4 | 1.4 |
| **ID07** | T_0_ | 11506852 | 6237006 | 5579816 | 14607754 | 7.7 | 1.5 | 1.5 |
|  | T_1_ | 13488396 | 7556022 | 6785654 | 12119617 | 7.8 | 1.5 | 1.5 |
|  | T_2_ | 2719358 | 2130292 | 1974080 | 1947283 | 18.6 | 12.9 | 12.8 |
|  | T_r_ | 3341222 | 2337280 | 2197732 | 2831960 | 19.3 | 13.4 | 13.2 |
| **ID08** | T_0_ | 3414512 | 2627626 | 2422062 | 9159723 | 20.8 | 13.4 | 13.3 |
|  | T_1_ | 3494272 | 2711854 | 2491954 | 13057918 | 20.8 | 13.7 | 13.5 |
|  | T_2_ | 3517608 | 2796508 | 2605726 | 14623431 | 20.4 | 12.9 | 12.8 |
|  | T_3_ | 3589664 | 2762582 | 2546480 | 25853706 | 19.9 | 12.8 | 12.8 |
|  | T_4_ | 24448286 | 13438312 | 11916370 | 84115023 | 7.6 | 1.4 | 1.5 |
|  | T_5_ | 25170624 | 14120722 | 12270064 | 90874336 | 8.6 | 1.6 | 1.7 |
|  | T_r_ | 24430366 | 13223710 | 11526652 | 95510093 | 11.0 | 1.6 | 1.7 |
| **ID09** | T_0_ | 14826088 | 8622104 | 7745014 | 15360391 | 14.1 | 2.1 | 2.4 |
|  | T_1_ | 14902416 | 8570462 | 7718352 | 13888186 | 11.0 | 1.5 | 1.6 |
|  | T_2_ | 13535454 | 7981384 | 7244140 | 14556900 | 11.5 | 1.8 | 1.9 |
|  | T_3_ | 13759582 | 7909534 | 7056098 | 12351933 | 13.9 | 1.4 | 1.5 |
|  | T_4_ | 12487382 | 7434634 | 6745396 | 12481210 | 9.6 | 1.3 | 1.4 |
|  | T_5_ | 12737506 | 7480214 | 6786382 | 10540991 | 8.7 | 1.7 | 1.9 |
| **ID10** | T_0_ | 3208494 | 2474762 | 2292602 | 3292755 | 25.1 | 15.1 | 15.2 |
|  | T_r_ | 3102428 | 2320616 | 2153290 | 2537931 | 27.5 | 18 | 17.9 |
| **ID11** | T_0_ | 18204124 | 10368368 | 8958570 | 11217720 | 17.4 | 2.0 | 2.1 |
|  | T_1_ | 13158440 | 7571622 | 6776442 | 14207231 | 13.2 | 2.3 | 2.5 |
|  | T_r_ | 13875366 | 8110020 | 7251286 | 19301513 | 14.6 | 2.1 | 2.2 |
| **ID12** | T_0_ | 12770968 | 7386952 | 6737000 | 6426407 | 8.3 | 1.8 | 1.9 |
|  | T_1_ | 15607302 | 8992036 | 8128510 | 9752515 | 13.0 | 1.9 | 2.1 |
|  | T_2_ | 24049012 | 11613658 | 10257394 | 6061974 | 8.2 | 2.2 | 2.3 |
|  | T_3_ | 9881544 | 7435018 | 7004394 | 1967723 | 20.1 | 13.5 | 13.3 |
|  | T_4_ | 27012740 | 13196308 | 11900228 | 8751174 | 9.0 | 2.2 | 2.3 |
|  | T_5_ | 10969912 | 8330434 | 7840942 | 2215764 | 22.9 | 16.2 | 16.0 |
|  | T_6_ | 11068846 | 8511612 | 8005918 | 3988359 | 21.6 | 14.8 | 14.5 |
| **ID13** | T_0_ | 8731652 | 6559786 | 6063422 | 1215374 | 22.0 | 15.5 | 15.3 |
|  | T_1_ | 26891540 | 13868692 | 12515282 | 8226669 | 7.4 | 1.9 | 2.0 |
|  | T_2_ | 30229438 | 15259654 | 13361940 | 20093673 | 20.7 | 2.0 | 2.2 |
|  | T_3_ | 8839098 | 6881286 | 6548898 | 24809803 | 9.3 | 2.0 | 2.0 |
|  | T_4_ | 8334154 | 6402290 | 6103528 | 13198015 | 6.8 | 1.4 | 1.4 |
|  | T_5_ | 14615238 | 11074464 | 10526460 | 13073852 | 6.9 | 1.3 | 1.3 |
|  | T_r_ | 8489302 | 6435552 | 6130230 | 18669832 | 6.9 | 1.3 | 1.3 |
| **ID14** | T_0_ | 7979466 | 5949584 | 5655470 | 9139353 | 7.0 | 1.3 | 1.3 |
|  | T_1_ | 9396736 | 6942324 | 6637768 | 8096443 | 6.3 | 1.4 | 1.5 |
|  | T_r_ | 8949404 | 6595336 | 6259016 | 8851002 | 7.4 | 2.1 | 2.2 |
| **ID15** | T_0_ | 7559500 | 149172 | 117084 | 251442 | 8.7 | 3.1 | 4.2 |
|  | T_1_ | 9327968 | 6739760 | 6319970 | 6385302 | 10.9 | 1.4 | 1.4 |
|  | T_2_ | 9798274 | 6905750 | 6416142 | 6722348 | 11.8 | 2.4 | 2.4 |
|  | T_r_ | 8986914 | 538706 | 414150 | 308797 | 11.3 | 6.5 | 9.8 |
| **ID16** | T_0_ | 7121754 | 152296 | 107430 | 506846 | 10.7 | 1.5 | 1.8 |
|  | T_1_ | 2247880 | 107636 | 81958 | 135213 | 11.5 | 5.3 | 9.1 |
|  | T_2_ | 4617386 | 109216 | 84574 | 200303 | 10.9 | 3.9 | 5.2 |
|  | T_3_ | 6724666 | 5004768 | 4756358 | 11313187 | 9.6 | 1.3 | 1.3 |
|  | T_r_ | 7847782 | 5784644 | 5452250 | 16169105 | 20.4 | 1.1 | 1.1 |
| **ID17** | T_0_ | 26182818 | 13156508 | 12024532 | 37507941 | 7.8 | 2.2 | 2.2 |
|  | T_r_ | 6189272 | 2102476 | 1597536 | 2185964 | 18.7 | 12.2 | 13.0 |
| **ID18** | T_0_ | 23015150 | 11256638 | 10221864 | 8237515 | 8.7 | 2.4 | 2.5 |
|  | T_1_ | 4641612 | 2431436 | 2201904 | 9441197 | 11.5 | 2.4 | 2.5 |
|  | T_r_ | 16906784 | 8213926 | 7161592 | 2973302 | 22.1 | 13.5 | 13.7 |
| **ID19** | T_0_ | 17277568 | 8745458 | 7710306 | 1248689 | 21.4 | 13.1 | 13.4 |
|  | T_r_ | 23165616 | 12772622 | 11391980 | 4678771 | 25.8 | 14.3 | 14.4 |
| **ID20** | T_0_ | 26458034 | 12969700 | 11601922 | 3977399 | 8.9 | 3.0 | 3.1 |
|  | T_1_ | 16051944 | 8074114 | 7226146 | 2344794 | 7.3 | 1.1 | 1.1 |
|  | T_r_ | 11407238 | 5860572 | 5286674 | 8284296 | 9.9 | 1.1 | 1.1 |
| **ID21** | T_0_ | 10666630 | 5910648 | 5411368 | 14051290 | 9.3 | 2.2 | 2.1 |
|  | T_1_ | 9346976 | 4863918 | 4413954 | 6421569 | 6.9 | 1.4 | 1.4 |
|  | T_2_ | 11592964 | 6169640 | 5610360 | 9139209 | 6.8 | 1.2 | 1.2 |
|  | T_3_ | 9113248 | 4803590 | 4361788 | 9471254 | 10.3 | 3.9 | 3.9 |
|  | T_4_ | 12512392 | 6746970 | 6076860 | 18422009 | 7.1 | 1.4 | 1.4 |
|  | T_r_ | 13090874 | 6516420 | 5726910 | 3475502 | 7.1 | 1.0 | 1.0 |
| **ID22** | T_0_ | 21205616 | 10419444 | 9457928 | 5450370 | 7.9 | 2.1 | 2.1 |
|  | T_1_ | 23780076 | 11546800 | 10404826 | 10705949 | 10.6 | 2.4 | 2.4 |
|  | T_2_ | 25853468 | 12344952 | 11093016 | 4775630 | 8.8 | 2.0 | 2.0 |

**Supplementary table 2B**: List of technical parameters supporting methylation analysis adopting proprietary bioinformatic pipeline of Avida Duo Methyl Reagent Kit (Avida Biomed).

*Abbreviations*: C (Cytosine); CHG (sequence where H represent any nucleotide except Guanine (A. C or T));
CHH (sequence where H represent any nucleotide except Guanine (A. C or T)); CpG (sequence where a Cytosine (C) is followed by a Guanine (G)); T_0_ (Baseline timepoint); T_1_ (first longitudinal timepoint); T_r_ (resistance timepoint).
