# Supplementary material for: Exploring genomic analysis and methylome profiling in longitudinal series of p.G12C KRAS mutated NSCLC patients treated with sotorasib
Source: J Liq Biopsy. 2026 Apr 27;12:100467. doi: 10.1016/j.jlb.2026.100467 (PMC13146550; doi:10.1016/j.jlb.2026.100467)
Supplement: Multimedia component 4 [file mmc4.docx]

| **ID Sample** | **Collection point** | **Valid partitions** | **Channel** | **Positive partitions** | **Concentration (cp/μl)** | **VAF** |
| --- | --- | --- | --- | --- | --- | --- |
| **ID01** | T_0_ | 25080 | FAM | 4 | 0.15 | 7.14 |
|  |  |  | HEX | 52 | 1.92 |  |
|  | T_1_ | 24837 | FAM | 0 | 0.00 | 0.00 |
|  |  |  | HEX | 58 | 2.16 |  |
|  | T_r_ | 24642 | FAM | 0 | 0.00 | 0.00 |
|  |  |  | HEX | 33 | 1.24 |  |
| **ID02** | T_0_ | 24721 | FAM | 326 | 12.27 | 56.54 |
|  |  |  | HEX | 251 | 9.43 |  |
|  | T_1_ | 24901 | FAM | 82 | 3.05 | 50.93 |
|  |  |  | HEX | 79 | 2.94 |  |
|  | T_r_ | 24998 | FAM | 599 | 22.42 | 75.21 |
|  |  |  | HEX | 199 | 7.39 |  |
| **ID03** | T_0_ | 24792 | FAM | 1 | 0.04 | 1.69 |
|  |  |  | HEX | 58 | 2.16 |  |
|  | T_1_ | 24597 | FAM | 0 | 0.00 | 0.00 |
|  |  |  | HEX | 24 | 0.90 |  |
|  | T_2_ | 24473 | FAM | 0 | 0.00 | 0.00 |
|  |  |  | HEX | 24 | 0.91 |  |
|  | T_r_ | 24209 | FAM | 0 | 0.00 | 0.00 |
|  |  |  | HEX | 32 | 1.22 |  |
| **ID04** | T_0_ | 24399 | FAM | 0 | 0.00 | 0.00 |
|  |  |  | HEX | 19 | 0.72 |  |
|  | T_1_ | 24767 | FAM | 0 | 0.00 | 0.00 |
|  |  |  | HEX | 205 | 7.68 |  |
| **ID05** | T_0_ | 24901 | FAM | 0 | 0.00 | 0.00 |
|  |  |  | HEX | 64 | 2.38 |  |
|  | T_1_ | 24456 | FAM | 0 | 0.00 | 0.00 |
|  |  |  | HEX | 67 | 2.54 |  |
|  | T_2_ | 24941 | FAM | 0 | 0.00 | 0.00 |
|  |  |  | HEX | 56 | 2.08 |  |
|  | T_3_ | 25299 | FAM | 0 | 0.00 | 0.00 |
|  |  |  | HEX | 66 | 2.41 |  |
|  | T_4_ | 24817 | FAM | 0 | 0.00 | 0.00 |
|  |  |  | HEX | 78 | 2.91 |  |
|  | T_5_ | 24773 | FAM | 0 | 0.00 | 0.00 |
|  |  |  | HEX | 56 | 2.09 |  |
|  | T_6_ | 25266 | FAM | 0 | 0.00 | 0.00 |
|  |  |  | HEX | 108 | 3.96 |  |
|  | T_7_ | 25154 | FAM | 1 | 0.04 | 1.58 |
|  |  |  | HEX | 62 | 2.28 |  |
| **ID06** | T_0_ | 24357 | FAM | 1 | 0.04 | 0.91 |
|  |  |  | HEX | 109 | 4.15 |  |
|  | T_1_ | 24282 | FAM | 0 | 0.00 | 0.00 |
|  |  |  | HEX | 64 | 2.44 |  |
|  | T_2_ | 24942 | FAM | 0 | 0.00 | 0.00 |
|  |  |  | HEX | 75 | 2.78 |  |
|  | T_r_ | 24783 | FAM | 0 | 0.00 | 0.00 |
|  |  |  | HEX | 105 | 3.92 |  |
| **ID07** | T_0_ | 24994 | FAM | 0 | 0.00 | 0.00 |
|  |  |  | HEX | 114 | 4.23 |  |
|  | T_1_ | 24917 | FAM | 1 | 0.04 | 1.30 |
|  |  |  | HEX | 76 | 2.82 |  |
|  | T_2_ | 24636 | FAM | 0 | 0.00 | 0.00 |
|  |  |  | HEX | 124 | 4.66 |  |
|  | T_r_ | 25186 | FAM | 6 | 0.22 | 3.08 |
|  |  |  | HEX | 188 | 6.92 |  |
| **ID08** | T_0_ | 25122 | FAM | 1 | 0.00 | 0.00 |
|  |  |  | HEX | 498 | 18.51 |  |
|  | T_1_ | 24557 | FAM | 0 | 0.00 | 0.00 |
|  |  |  | HEX | 874 | 33.49 |  |
|  | T_2_ | 24577 | FAM | 0 | 0.00 | 0.00 |
|  |  |  | HEX | 679 | 25.89 |  |
|  | T_3_ | 24586 | FAM | 0 | 0.00 | 0.00 |
|  |  |  | HEX | 1853 | 72.42 |  |
|  | T_4_ | 25032 | FAM | 0 | 0.00 | 0.00 |
|  |  |  | HEX | 1106 | 41.76 |  |
|  | T_5_ | 24728 | FAM | 0 | 0.00 | 0.00 |
|  |  |  | HEX | 936 | 35.66 |  |
|  | T_r_ | 25209 | FAM | 2 | 0.07 | 0.15 |
|  |  |  | HEX | 1270 | 47.77 |  |
| **ID09** | T_0_ | 25367 | FAM | 4 | 0.15 | 2.69 |
|  |  |  | HEX | 144 | 5.26 |  |
|  | T_1_ | 25038 | FAM | 0 | 0.00 | 0.00 |
|  |  |  | HEX | 147 | 5.44 |  |
|  | T_2_ | 24685 | FAM | 0 | 0.00 | 0.00 |
|  |  |  | HEX | 162 | 6.09 |  |
|  | T_3_ | 25232 | FAM | 0 | 0.00 | 0.00 |
|  |  |  | HEX | 116 | 4.26 |  |
|  | T_4_ | 25081 | FAM | 0 | 0.00 | 0.00 |
|  |  |  | HEX | 292 | 10.82 |  |
|  | T_5_ | 25182 | FAM | 0 | 0.00 | 0.00 |
|  |  |  | HEX | 130 | 4.78 |  |
| **ID10** | T_0_ | 23232 | FAM | 29 | 1.15 | 15.56 |
|  |  |  | HEX | 157 | 6.27 |  |
|  | T_r_ | 25189 | FAM | 39 | 1.43 | 10.90 |
|  |  |  | HEX | 317 | 11.70 |  |
| **ID11** | T_0_ | 25346 | FAM | 29 | 1.06 | 17.76 |
|  |  |  | HEX | 134 | 4.90 |  |
|  | T_1_ | 25052 | FAM | 9 | 0.33 | 5.28 |
|  |  |  | HEX | 161 | 5.96 |  |
|  | T_r_ | 25100 | FAM | 40 | 1.47 | 9.94 |
|  |  |  | HEX | 360 | 13.35 |  |
| **ID12** | T_0_ | 25159 | FAM | 3 | 0.11 | 4.54 |
|  |  |  | HEX | 63 | 2.32 |  |
|  | T_1_ | 25306 | FAM | 0 | 0.00 | 0.00 |
|  |  |  | HEX | 100 | 3.66 |  |
|  | T_2_ | 24992 | FAM | 0 | 0.00 | 0.00 |
|  |  |  | HEX | 87 | 3.22 |  |
|  | T_3_ | 24865 | FAM | 0 | 0.00 | 0.00 |
|  |  |  | HEX | 146 | 5.44 |  |
|  | T_4_ | 25143 | FAM | 0 | 0.00 | 0.00 |
|  |  |  | HEX | 142 | 5.23 |  |
|  | T_5_ | 24390 | FAM | 0 | 0.00 | 0.00 |
|  |  |  | HEX | 95 | 3.61 |  |
|  | T_6_ | 24923 | FAM | 0 | 0.00 | 0.00 |
|  |  |  | HEX | 220 | 8.19 |  |
| **ID13** | T_0_ | 25190 | FAM | 0 | 0.00 | 0.00 |
|  |  |  | HEX | 102 | 3.75 |  |
|  | T_1_ | 25012 | FAM | 0 | 0.00 | 0.00 |
|  |  |  | HEX | 114 | 4.22 |  |
|  | T_2_ | 24955 | FAM | 0 | 0.00 | 0.00 |
|  |  |  | HEX | 186 | 6.91 |  |
|  | T_3_ | 25014 | FAM | 0 | 0.00 | 0.00 |
|  |  |  | HEX | 361 | 13.44 |  |
|  | T_4_ | 25172 | FAM | 0 | 0.00 | 0.00 |
|  |  |  | HEX | 129 | 4.75 |  |
|  | T_5_ | 25152 | FAM | 2 | 0.07 | 1.49 |
|  |  |  | HEX | 132 | 4.86 |  |
|  | T_r_ | 24791 | FAM | 3 | 0.11 | 1.42 |
|  |  |  | HEX | 208 | 7.79 |  |
| **ID14** | T_0_ | 24845 | FAM | 0 | 0.00 | 0.00 |
|  |  |  | HEX | 106 | 3.95 |  |
|  | T_1_ | 25028 | FAM | 0 | 0.00 | 0.00 |
|  |  |  | HEX | 93 | 3.44 |  |
|  | T_r_ | 25203 | FAM | 1 | 0.04 | 0.76 |
|  |  |  | HEX | 130 | 4.78 |  |
| **ID15** | T_0_ | 24612 | FAM | 0 | 0.00 | 0.00 |
|  |  |  | HEX | 51 | 1.92 |  |
|  | T_1_ | 24997 | FAM | 0 | 0.00 | 0.00 |
|  |  |  | HEX | 68 | 2.52 |  |
|  | T_2_ | 25385 | FAM | 0 | 0.00 | 0.00 |
|  |  |  | HEX | 61 | 2.22 |  |
|  | T_r_ | 25075 | FAM | 0 | 0.00 | 0.00 |
|  |  |  | HEX | 55 | 2.03 |  |
| **ID16** | T_0_ | 24714 | FAM | 0 | 0.00 | 0.00 |
|  |  |  | HEX | 163 | 6.12 |  |
|  | T_1_ | 25175 | FAM | 0 | 0.00 | 0.00 |
|  |  |  | HEX | 121 | 4.45 |  |
|  | T_2_ | 25173 | FAM | 1 | 0.04 | 1.22 |
|  |  |  | HEX | 81 | 2.98 |  |
|  | T_3_ | 24535 | FAM | 76 | 2.87 | 40.19 |
|  |  |  | HEX | 113 | 4.27 |  |
|  | T_r_ | 24857 | FAM | 436 | 16.35 | 76.74 |
|  |  |  | HEX | 133 | 4.96 |  |
| **ID17** | T_0_ | 25210 | FAM | 2 | 0.07 | 0.37 |
|  |  |  | HEX | 537 | 19.90 |  |
|  | T_r_ | 25363 | FAM | 1 | 0.04 | 0.37 |
|  |  |  | HEX | 266 | 9.7 |  |
| **ID18** | T_0_ | 25259 | FAM | 2 | 0.07 | 1.34 |
|  |  |  | HEX | 147 | 5.39 |  |
|  | T_1_ | 25102 | FAM | 0 | 0.00 | 0.00 |
|  |  |  | HEX | 156 | 5.76 |  |
|  | T_r_ | 25023 | FAM | 23 | 0.85 | 20.89 |
|  |  |  | HEX | 87 | 3.22 |  |
| **ID19** | T_0_ | 25021 | FAM | 2 | 0.07 | 2.50 |
|  |  |  | HEX | 78 | 2.89 |  |
|  | T_r_ | 25293 | FAM | 137 | 5.02 | 26.05 |
|  |  |  | HEX | 387 | 14.25 |  |
| **ID20** | T_0_ | 25237 | FAM | 0 | 0.00 | 0.00 |
|  |  |  | HEX | 69 | 2.53 |  |
|  | T_1_ | 24951 | FAM | 0 | 0.00 | 0.00 |
|  |  |  | HEX | 67 | 2.49 |  |
|  | T_r_ | 24404 | FAM | 103 | 3.91 | 48.13 |
|  |  |  | HEX | 111 | 4.21 |  |
| **ID21** | T_0_ | 24605 | FAM | 12 | 0.45 | 7.43 |
|  |  |  | HEX | 149 | 5.61 |  |
|  | T_1_ | 24706 | FAM | 1 | 0.04 | 1.28 |
|  |  |  | HEX | 77 | 2.89 |  |
|  | T_2_ | 24893 | FAM | 1 | 0.04 | 1.26 |
|  |  |  | HEX | 78 | 2.90 |  |
|  | T_3_ | 24908 | FAM | 2 | 0.07 | 1.25 |
|  |  |  | HEX | 157 | 5.84 |  |
|  | T_4_ | 24848 | FAM | 2 | 0.07 | 1.18 |
|  |  |  | HEX | 167 | 6.23 |  |
|  | T_r_ | 25287 | FAM | 23 | 0.84 | 12.54 |
|  |  |  | HEX | 160 | 5.87 |  |
| **ID22** | T_0_ | 25096 | FAM | 0 | 0.00 | 0.00 |
|  |  |  | HEX | 62 | 2.29 |  |
|  | T_1_ | 24748 | FAM | 0 | 0.00 | 0.00 |
|  |  |  | HEX | 146 | 5.47 |  |
|  | T_2_ | 24092 | FAM | 0 | 0.00 | 0.00 |
|  |  |  | HEX | 45 | 1.73 |  |

**Supplementary table 3:** technical parameters supporting *KRAS* p.G12C detection by dPCR (Digital LightCycler, Roche) system on a longitudinal series of plasma samples.

*Abbreviations*: cp/μl (copies per microliter); dPCR (digital Polymerase Chain Reaction); VAF (variant allele frequency); T_0_ (Baseline timepoint); T_1_ (first longitudinal timepoint); T_r_ (resistance timepoint).
