# Supplementary material for: Exploring genomic analysis and methylome profiling in longitudinal series of p.G12C KRAS mutated NSCLC patients treated with sotorasib
Source: J Liq Biopsy. 2026 Apr 27;12:100467. doi: 10.1016/j.jlb.2026.100467 (PMC13146550; doi:10.1016/j.jlb.2026.100467)
Supplement: Multimedia component 5 [file mmc5.docx]

| **ID Sample** | **Collection point** | **Methylation Index  (Alyssa Reporter)** | **Methylation Index  (SeqOne)** |  | **ID Sample** | **Collection point** | **Methylation Index  (Alyssa Reporter)** | **Methylation Index  (SeqOne)** |
| --- | --- | --- | --- | --- | --- | --- | --- | --- |
| **ID01** | T_0_ | 1.7 | 1.5 |  |  | T_6_ | 0.3 | 0.3 |
|  | T_1_ | 0.4 | 0.4 |  | **ID13** | T_0_ | 0.1 | 0.0 |
|  | T_r_ | 0.3 | 0.4 |  |  | T_1_ | 0.3 | 0.3 |
| **ID02** | T_0_ | 12.3 | 12.5 |  |  | T_2_ | 0.9 | 0.9 |
|  | T_1_ | 4.6 | 4.2 |  |  | T_3_ | 0.5 | 0.5 |
|  | T_r_ | 7.4 | 8.1 |  |  | T_4_ | 0.5 | 0.6 |
| **ID03** | T_0_ | 14.5 | 13.4 |  |  | T_5_ | 1.2 | 1.3 |
|  | T_1_ | 1.5 | 1.4 |  |  | T_r_ | 2.0 | 2.1 |
|  | T_2_ | 2.4 | 2.2 |  | **ID14** | T_0_ | 0.1 | 0.2 |
|  | T_r_ | 5.6 | 5.6 |  |  | T_1_ | 0.3 | 0.4 |
| **ID04** | T_0_ | 0.2 | 0.3 |  |  | T_r_ | 0.3 | 0.3 |
|  | T_1_ | 1.3 | 1.5 |  | **ID15** | T_0_ | 0.6 | 0.8 |
| **ID05** | T_0_ | 0.5 | 0.5 |  |  | T_1_ | 0.2 | 0.2 |
|  | T_1_ | 0.2 | 0.3 |  |  | T_2_ | 0.7 | 0.8 |
|  | T_2_ | 0.3 | 0.3 |  |  | T_r_ | 0.3 | 0.3 |
|  | T_3_ | 0.6 | 0.6 |  | **ID16** | T_0_ | 0.9 | 1.1 |
|  | T_4_ | 0.7 | 0.7 |  |  | T_1_ | 0.4 | 0.2 |
|  | T_5_ | 0.7 | 0.6 |  |  | T_2_ | 3.0 | 3.0 |
|  | T_6_ | 0.6 | 0.6 |  |  | T_3_ | 12.9 | 13.0 |
|  | T_7_ | 0.4 | 0.5 |  |  | T_r_ | 55.8 | 56.2 |
| **ID06** | T_0_ | 0.4 | 0.4 |  | **ID17** | T_0_ | 0.4 | 0.4 |
|  | T_1_ | 0.1 | 0.2 |  |  | T_r_ | 0.3 | 0.3 |
|  | T_2_ | 0.4 | 0.3 |  | **ID18** | T_0_ | 0.6 | 0.6 |
|  | T_r_ | 0.1 | 0.1 |  |  | T_1_ | 0.2 | 0.2 |
| **ID07** | T_0_ | 0.3 | 0.4 |  |  | T_r_ | 0.5 | 0.5 |
|  | T_1_ | 0.2 | 0.3 |  | **ID19** | T_0_ | 0.7 | 0.8 |
|  | T_2_ | 0.2 | 0.2 |  |  | T_r_ | 1.8 | 1.9 |
|  | T_r_ | 0.1 | 0.1 |  | **ID20** | T_0_ | 0.4 | 0.3 |
| **ID08** | T_0_ | 0.1 | 0.1 |  |  | T_1_ | 0.7 | 0.9 |
|  | T_1_ | 0.1 | 0.1 |  |  | T_r_ | 11.0 | 11.5 |
|  | T_2_ | 0.1 | 0.2 |  | **ID21** | T_0_ | 3.3 | 3.5 |
|  | T_3_ | 0.1 | 0.1 |  |  | T_1_ | 0.5 | 0.5 |
|  | T_4_ | 0.3 | 0.4 |  |  | T_2_ | 0.4 | 0.5 |
|  | T_5_ | 0.4 | 0.5 |  |  | T_3_ | 0.3 | 0.5 |
|  | T_r_ | 0.7 | 0.8 |  |  | T_4_ | 0.7 | 0.6 |
| **ID09** | T_0_ | 2.1 | 2.2 |  |  | T_r_ | 2.5 | 2.9 |
|  | T_1_ | 0.2 | 0.3 |  | **ID22** | T_0_ | 0.8 | 0.8 |
|  | T_2_ | 0.2 | 0.2 |  |  | T_1_ | 0.8 | 0.9 |
|  | T_3_ | 0.3 | 0.3 |  |  | T_2_ | 0.4 | 0.5 |
|  | T_4_ | 0.1 | 0.1 |  |  |  |  |  |
|  | T_5_ | 0.4 | 0.5 |  |  |  |  |  |
| **ID10** | T_0_ | 1.8 | 2.1 |  |  |  |  |  |
|  | T_r_ | 0.9 | 1.0 |  |  |  |  |  |
| **ID11** | T_0_ | 14.8 | 15.8 |  |  |  |  |  |
|  | T_1_ | 5.4 | 5.5 |  |  |  |  |  |
|  | T_r_ | 7.1 | 7.5 |  |  |  |  |  |
| **ID12** | T_0_ | 0.5 | 0.5 |  |  |  |  |  |
|  | T_1_ | 0.3 | 0.3 |  |  |  |  |  |
|  | T_2_ | 0.1 | 0.1 |  |  |  |  |  |
|  | T_3_ | 0.1 | 0.0 |  |  |  |  |  |
|  | T_4_ | 0.5 | 0.5 |  |  |  |  |  |
|  | T_5_ | 0.1 | 0.1 |  |  |  |  |  |

**Supplementary table 4***:* Methylation Index processed with two different bioinformatic pipelines (Alyssa Reporter (Agilent, CA, USA) and SeqOne Platform (SeqOne Genomics, France) in longitudinal series of plasma samples.

*Abbreviations*: T_0_ (Baseline timepoint); T_n_ (longitudinal timepoints); T_r_ (resistance timepoint)
